# Abundance and Diversity of Bacterial Nitrifiers and Denitrifiers and Their Functional Genes in Tannery Wastewater Treatment Plants Revealed by High-Throughput Sequencing

**DOI:** 10.1371/journal.pone.0113603

**Published:** 2014-11-24

**Authors:** Zhu Wang, Xu-Xiang Zhang, Xin Lu, Bo Liu, Yan Li, Chao Long, Aimin Li

**Affiliations:** 1 State Key Laboratory of Pollution Control and Resource Reuse, School of the Environment, Nanjing University, Nanjing, China; 2 Research Institute of Nanjing University in Lianyungang, Lianyungang, China; CSIR, India

## Abstract

Biological nitrification/denitrification is frequently used to remove nitrogen from tannery wastewater containing high concentrations of ammonia. However, information is limited about the bacterial nitrifiers and denitrifiers and their functional genes in tannery wastewater treatment plants (WWTPs) due to the low-throughput of the previously used methods. In this study, 454 pyrosequencing and Illumina high-throughput sequencing, combined with molecular methods, were used to comprehensively characterize structures and functions of nitrification and denitrification bacterial communities in aerobic and anaerobic sludge of two full-scale tannery WWTPs. Pyrosequencing of 16S rRNA genes showed that *Proteobacteria* and *Synergistetes* dominated in the aerobic and anaerobic sludge, respectively. Ammonia-oxidizing bacteria (AOB) *amoA* gene cloning revealed that *Nitrosomonas europaea* dominated the ammonia-oxidizing community in the WWTPs. Metagenomic analysis showed that the denitrifiers mainly included the genera of *Thauera*, *Paracoccus*, *Hyphomicrobium*, *Comamonas* and *Azoarcus*, which may greatly contribute to the nitrogen removal in the two WWTPs. It is interesting that AOB and ammonia-oxidizing archaea had low abundance although both WWTPs demonstrated high ammonium removal efficiency. Good correlation between the qPCR and metagenomic analysis is observed for the quantification of functional genes *amoA*, *nirK*, *nirS* and *nosZ*, indicating that the metagenomic approach may be a promising method used to comprehensively investigate the abundance of functional genes of nitrifiers and denitrifiers in the environment.

## Introduction

Tannery wastewater is characterized by high contents of nitrogen, organic compounds and salt ions [1],[2], and the complexity of the wastewater components can affect microbial nitrification/denitrification processes that have been frequently used for nitrogen removal in wastewater treatment plants (WWTPs). Nitrification, the biological oxidation of ammonium to nitrite and nitrate, is carried out in two sequential steps via several phylogenetically distinct groups of microorganisms: ammonia-oxidizing archaea (AOA), ammonia-oxidizing bacteria (AOB) and nitrite-oxidizing bacteria (NOB) [3]. Denitrification consists of consecutive reactions including transformation of nitrate or nitrite into gaseous forms (N_2_ or N_2_O) by different groups of bacteria (nitrifiers and denitrifiers) [4].

Due to the technological difficulty in isolation and culture of bacterial nitrifiers and denitrifiers, molecular methods have been widely used to analyze diversity and abundance of microorganisms involved in nitrification/denitrification in various environments, such as soil [5] and activated sludge [4]. Previous studies have investigated the microbial community of activated sludge in tannery WWTPs through 16S rRNA gene amplification and DNA sequencing [1],[6]. However, information about abundance and diversity of nitrifying and denitrifying bacteria in tannery WWTPs is limited due to the low throughput of those techniques.

Recently, high-throughput sequencing techniques, such as 454 pyrosequencing and Illumina sequencing methods, have shown great advantages on microbial community analysis due to their unprecedented sequencing depth, which have been used to deeply explore microbial communities in drinking water [7], soil [8] and WWTPs [9]–[11]. However, the metagenomic methods have not been used for comprehensive insights into the communities of nitrifies and denitrifies in tannery WWTPs.

In this study, 454 pyrosequencing of 16S rRNA gene was conducted to determine the bacterial community structure of activated sludge sampled from two full-scale tannery WWTPs, and Illumina high-throughput sequencing was used to comprehensively investigate functional genes involved in the bacterial nitrification and denitrification. To confirm the metagenomic results, quantitative real time PCR (qPCR) was used to quantify the functional genes encoding subunits of the ammonia monooxygenase (*amoA*), nitrite reductases (*nirK* and *nirS*) and nitrous oxide reductase (*nosZ*). Since ammonia oxidation is a rate-limiting step in nitrification, the *amoA* gene was chosen for genetic diversity analysis using DNA cloning and sequencing technology. The results of this study may help to extend our knowledge about the microbial nitrification and denitrification processes and their underlying biological mechanisms for industrial wastewater treatment.

## Materials and Methods

### Sludge sampling and DNA extraction

In this study, activated sludge samples were separately collected from two full-scale WWTPs treating tannery wastewater of Boao Leather Industry Co., Ltd. (WWTPA geographically located in Xiangcheng City, Henan Province, China) and Yige Leather Industry Co., Ltd. (WWTPB geographically located in Zhecheng County, Henan Province, China). We would like to state that the two companies have approved this study which did not involve endangered or protected species. The two WWTPs basically involve a biological treatment system preceded by preliminary treatment (homogenization, chemical coagulation and primary settling). As described in our previous study [12], the biological treatment system of WWTPA was composed of an up-flow anaerobic sludge reactor (UASB) and an integrated anoxic/oxic (A/O) reactor, while WWTPB was composed of an oxidation ditch and an integrated anoxic/oxic (A/O) reactor. Relevant operational parameters about the two biological systems are shown in Table S1. Although the two WWTPs differed in the influent water quality and operational conditions, they both achieved high ammonia removal efficiencies (>97%). Thus, we chose the two WWTPs to explore the functional nitrifiers and denitrifiers responsible for the high ammonia removal. Four sludge samples were collected from the two WWTPs, including one anaerobic sludge sample (A-A) from the UASB and one aerobic sludge sample (A-O) from the last aerobic tank of the A/O reactor in WWTPA, and two aerobic sludge samples separately collected from the oxidation ditch (B-D) and the last aerobic tank of the A/O reactor (B-O) in WWTPB. The sludge samples were fixed with 50% ethanol (v/v) on site before transporting to laboratory for DNA extraction. The fixed sludge was centrifuged at 4,000 rpm for 10 min to collect approximately 200 mg of the pellet for total genomic DNA extraction with the FastDNA Soil Kit (MP Biomedicals, CA, USA). The concentration and quality of the extracted DNA were determined with microspectrophotometry (NanoDrop ND-1000, NanoDrop Technologies, Willmington, DE, USA).

### PCR amplification, cloning, sequencing and phylogenetic analysis of amoA gene

PCR of AOA and AOB *amoA* genes was conducted by using the primers listed in Table S2. The 30-µL reaction mixture contained 3 µL of 10×buffer, 1.8 µL of 25 mM MgCl_2_, 1.2 µL of 10 mM dNTP mixture, 0.2 µL of Ex Taq polymerase (TaKaRa, Japan), 0.3 µL of each primer (10 µM) and 20–50 ng of genomic DNA. The PCR conditions were initial denaturation at 95°C for 3 min, followed by 35 cycles of 94°C for 60 s, 56°C (for AOB *amoA*) or 53°C (for AOA *amoA*) for 45 s, and 72°C for 60 s; with a final extension at 72°C for 10 min. PCR products were analyzed by gel electrophoresis using 1% (w/v) agarose in 1×TAE buffer.

PCR products were purified by using DNA Fragment Purification Kit (TaKaRa, Japan). The purified PCR products were cloned using pMD19-T Vector (TaKaRa, Japan). An AOB *amoA* gene clone library was constructed for each of the four sludge samples; however, AOA *amoA* gene library was constructed for only sample A-A since the other samples failed to generate PCR products. About 20 clones in each clone library were randomly selected for DNA sequencing. The obtained *amoA* gene sequences were aligned and the Jukes-Cantor distances between subsequent pairs of sequences were calculated with DNADIST from the PHYLIP package (http://www.phylip.com/), and operational taxonomic units (OTUs) were defined with a distance cut-off of 3% using the DOTUR program [13]. In order to construct the phylogenetic tree, one representative sequence in each OTU was selected and aligned with the reference sequences from National Center for Biotechnology Information (NCBI) (http://www.ncbi.nlm.nih.gov/). The neighbor-joining phylogenetic trees of AOB and AOA *amoA* genes sequences were separately created by MEGA 5.1 software (http://www.megasoftware.net/).

### qPCR

AOB and AOA *amoA* genes were used as molecular markers to determine the abundance of the nitrifiers. Meanwhile, the abundance of denitrifying bacteria was investigated by quantifying the genes *nirK*, *nirS* and *nosZ*. qPCR was performed in Corbett Real-Time PCR Machine with the Rotor-Gene 6000 Series Software 1.7 (QIAGEN, the Netherlands) using SYBR Green method. The 20-µL PCR mixture contained 10 µL of SYBR Premix Ex Taq Super Mix (TaKaRa Japan), 0.2 µL of each primer (10 µM) (Table S2), 8 µL of template DNA corresponding to 40 ng of total DNA and 1.6 µL of ddH_2_O. The reaction was initially denatured at 95°C for 3 min, followed by 40 cycles of denaturation at 95°C for 20 s, annealing at the given temperatures (Table S2) for 20 s and extension at 72°C for 40 s.

To determine gene abundance in one ng of extracted DNA, all targeted genes were cloned to plasmids following the method recommended by Zhang and Zhang [14] to generate qPCR standard curves. In order to correct for potential variations in DNA extraction efficiencies, eubacterial 16S rRNA genes were also quantified using the method recommended by Lopez-Gutierrez et al. [15]. All samples and standards were analyzed in triplicate, and the specificity of qPCR products was checked by melt curves observation and agarose electrophoresis. PCR efficiency ranged from 85.9% to 105.5% with R^2^ values over 0.991 for all calibration curves.

### 454 Pyrosequencing and Illumina high-throughput sequencing

Pyrosequencing and Illumina sequencing were conducted at Beijing Genome Institute (Shenzhen, China). For pyrosequencing, the bacterial DNA was amplified with a set of primers targeting the hypervariable V3-V4 region (about 460 bp) of the 16S rRNA gene. The forward primer was V3F (5'-ACTCCTACGGGAG GCAGCAG-3') and the reverse primer was V4R (5'-TACNVGGGTATCTAATCC-3'). Equal amounts of purified amplicon products bearing individual 10 nucleotide barcode from different samples were mixed for pyrosequencing on the Roche 454 FLX Titanium platform (Roche, USA).

DNA samples (10 µg each) were subject to high-throughput sequencing using Illumina Hiseq 2000 (Illumina, USA). A library consisting of 180-bp DNA fragment sequences was constructed according to the manufacturer's instructions before DNA sequencing. The strategy “Index 101 PE” (Paired End sequencing, 101-bp reads and 8-bp index sequence) was used for the Illumina sequencing, generating nearly equal amount of data for each sample. Low quality reads were removed following the method recommended by Shi et al. [7]. The 100-bp clean reads (about 1.0 Gb per each sample) were used for subsequent metagenomic analysis.

### Bioinformatic analysis on pyrosequencing datasets

After pyrosequencing, downstream sequence analyses were performed using the Ribosomal Database Project (RDP) (http://rdp.cme.msu.edu/) [16] and Mothur (http://www.mothur.org/) [17] following the method recommended by Zhang et al. [18]. All the raw reads were assigned to the designated sample based on their nucleotide barcode with the Pyrosequencing Pipeline Initial Process of the RDP, which also trimmed off the adapters and barcodes and removed sequences containing ambiguous ‘N’ or shorter than 200 bps. All samples were denoised individually using Mothur's ‘pre.cluster’ command to remove the erroneous sequences due to pyrosequencing errors. PCR chimeras were filtered out using the ‘chimera.slayer’ command in Mothur platform. The reads flagged as chimeras was extracted out using a self-written Python script, and those being assigned to any known genus with 90% confidence using the RDP Classifier were merged with the non-chimera reads.

Taxonomic assignment of the sequences was conducted individually for each sample using the RDP Classifier with a bootstrap cutoff of 50% [9],[18]. Unexpected archaeal sequences were manually removed before biodiversity analysis. After denoising, filtering out chimeras and removing the archaeal sequences, 6,471∼13,727 effective pyrosequencing reads were generated for each of the four sludge samples (Table S3). For fair comparison, the library size of each sample was normalized to the same sequencing depth (6,471 reads) by randomly removing the redundant reads. Richness and diversity indices including OTUs number and Shannon's diversity index, as well as rarefaction curves, were calculated using Mothur.Cluster analysis and principal component analysis (PCA) of the microbial community structures of the different samples were conducted with PAleontological STatistics software (PAST, version 3.01) [19] and R (version 3.0.1) [20], respectively.

### Bioinformatic analysis on Illumina sequencing datasets

The Illumina sequencing datasets were functionally annotated using the Clusters of Orthologous Groups (COG) [21] database in MG-RAST [22] with default parameters. In order to quantify the abundance of functional genes involved in nitrification and denitrification in the four samples, sequences of *amoA*, *nirK*, *nirS* and *nosZ* deposited in NCBI Nucleotide database (http://www.ncbi.nlm.nih.gov/nuccore/) were downloaded to construct a local database. After removing redundancies, the local database contained 17,483 sequences of AOA *amoA* gene, 12,211 sequences of AOB *amoA* gene, 5,716 sequences of *nirK* gene, 7,615 sequences of *nirS* gene and 5,436 sequences of *nosZ* gene. BLASTn was used to align all the sequencing reads against the local database, and a read was identified as *amoA*, *nirK*, *nirS* or *nosZ* if the BLAST hit (E-value cutoff at 10^−5^) had a nucleotide sequence identity of above 90% over an alignment of at least 50 bp [10].

Additionally, the annotated reads were extracted by using a self-written Python script and then assigned to specific bacteria at the genus level by BLASTX against NCBI-nr database at an E-value of 1E-5. The BLASTX results were visualized with MEGAN (http://ab.inf.uni-tuebingen.de/software/megan/) at a threshold of bitscore>50 [10].

### Accession numbers

The sequences of *amoA* for cloning library construction have been deposited in GenBank under accession numbers KF720406 to KF720513. The pyrosequencing datasets have been deposited into the NCBI Short Reads Archive Database (accession number: SRX396800). The Illumina metagenomic datasets are available at MG-RAST under accession numbers 4494863.3 (A-A), 4494888.3 (A-O), 4494854.3 (B-D) and 4494855.3 (B-O).

## Results and Discussion

### Biodiversity and functions of microbial communities of the WWTPs

OTUs and Shannon index analysis based on 16S rRNA gene pyrosequencing showed that the sample A-O from WWTPA had the richest biodiversity, followed by the anaerobic sludge sample A-A from the same WWTP (Table S3). This result is confirmed by the rarefaction curves (Figure S1) indicating that WWTPA samples had richer biodiversity than WWTPB. The biodiversity divergences between the two WWTPs may result from the differences in influent wastewater composition and biological treatment processes (Table S1). For example, WWTPB had higher levels of sodium salt than WWTPA in influent wastewater (Table S1), and it is known that that salinity is an important factor regulating and reducing bacterial diversity [23],[24]. In this study, the Good's coverage of each sample was above 0.93 and 0.96 at cutoff levels of 3% and 5%, respectively (Table S3), which indicated that the sequences generated at this sequencing depth could well represent the bacterial communities of the four sludge samples.

**Figure 1:**
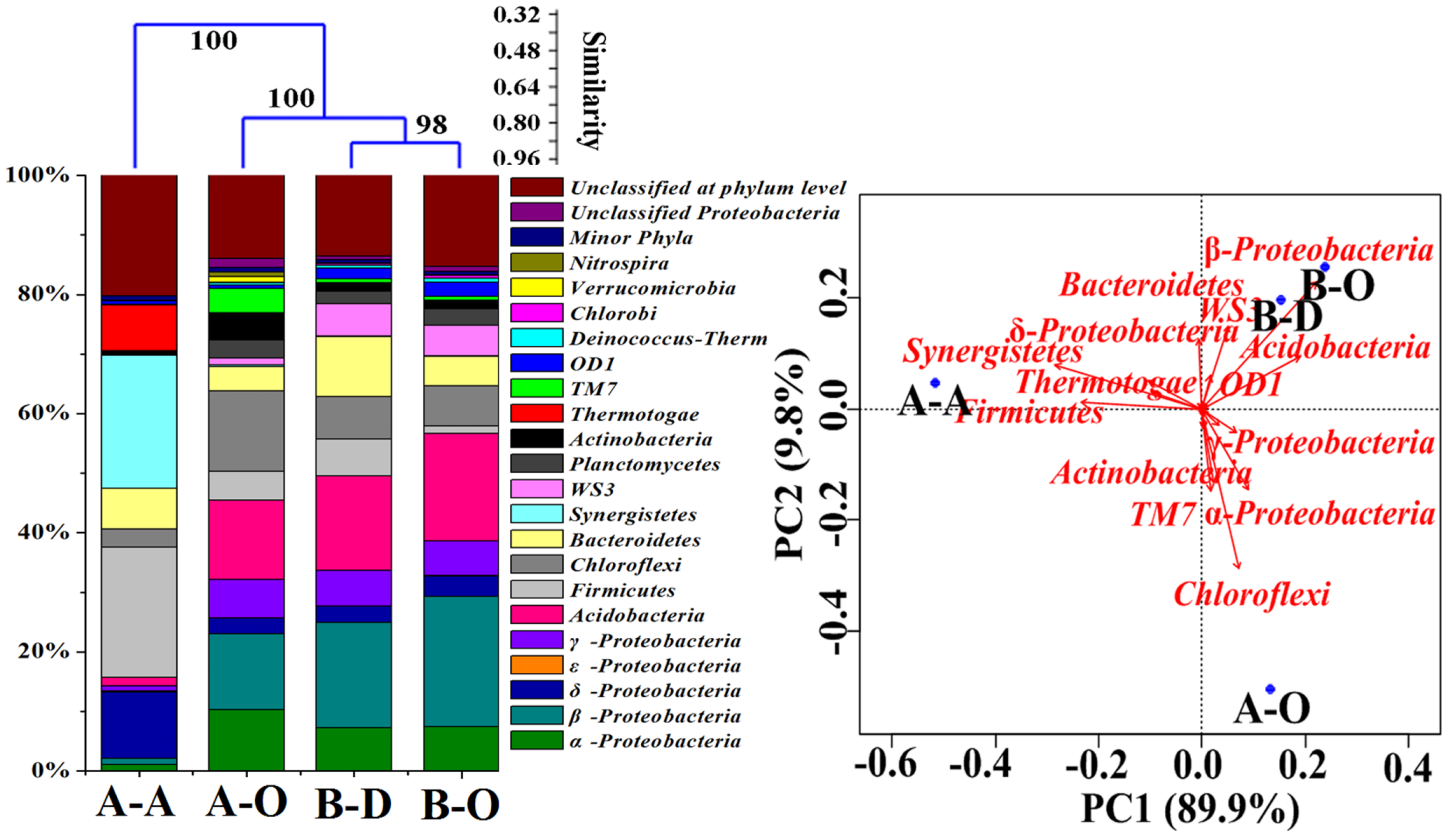
Abundance and distribution of different phyla and classes in *Proteobacteria* in the four sludge samples. Taxa shown occurred in at least one sample with abundance over 1%. Minor phyla refer to the taxa with their maximum abundance <1% in each sample. The effective pyrosequencing reads (6,471 sequences) were classified using RDP Classifier at a confidence threshold of 50%. Cluster analysis was conducted based on a distance matrix computed with Bray–Curtis similarity of four samples. Principal component analysis was conducted based on the phylum abundance using R (version 3.0.1).

Over 30% of the pyrosequencing reads from all samples could not be assigned to any taxa at genus level (Figure S2), which is comparable to the proportions of unclassified sequences in 14 sewage sludge samples reported by Zhang et al. [18] who used V4 region for 454 pyrosequencing. Cluster analysis showed that bacterial community structure of anaerobic sludge was divergent from those of the three aerobic sludge samples, and PCA revealed that the different samples harbored characteristic bacterial communities at phylum level (Figure 1). Samples B-D and B-O, two aerobic sludge samples which collected from WWTPB, were grouped together. However, bacterial community structures of aerobic sludge (A-O) and anaerobic sludge (A-A) of WWTPA were clearly separated, both of which were dramatically different from those of WWTPB. The difference in wastewater quality may result in the divergence of the aerobic sludge communities of the two WWTPs, and available oxygen is considered a crucial factor contributing to the difference between the aerobic and anaerobic sludge samples [25],[26].

Statistical analysis showed that 22.38% and 21.90% of the sequences in anaerobic sludge sample A-A were assigned into *Synergistetes* and *Firmicutes*, respectively, while *Proteobacteria* were the dominant phylum in the three aerobic sludge samples, accounting for about 33.64%, 34.31%, and 39.42% in samples A-O, B-D, and B-O, respectively (Figure 1). *Synergistetes* is known to have low abundance in aerobic sludge, but usually dominate in UASB reactors treating brewery wastewater [27] and tannery wastewater [1].

**Figure 2:**
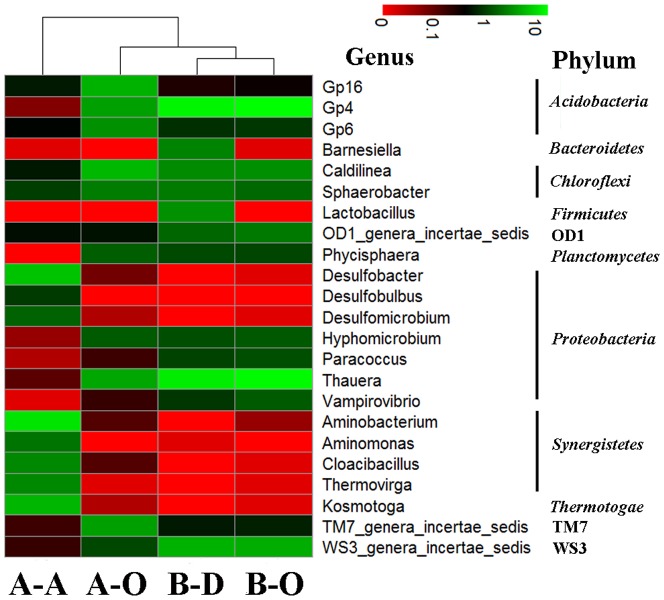
Heat map illustrating the abundance of all the major genera (with relative abundance over 1% in at least one sample). The color intensity (log scale) in each panel indicates the relative abundance of the genus in each sample.

Bacterial diversity and abundance were also analyzed more specifically at the genus level (Figure 2, Table S4). Cluster analysis also showed that the bacterial community structure of the anaerobic sludge was dramatically different from those of the aerobic sludge samples (Figure 2). Similar to the results obtained at phylum level, oxygen concentration is an important factor shaping microbial community structures in WWTPs, and may make huge contributions to the difference at genus level. It was found that anaerobes such as *Aminobacterium* (11.47%), *Desulfobacter* (6.94%) and *Kosmotoga* (5.75%) dominated in the anaerobic sludge sample A-A, while had relative lower abundance in the three aerobic sludge samples (<0.15%). Genus *Aminobacterium* belongs to the phylum *Synergistetes*, which is known as amino acids degraders [28]. Other genera in *Synergistetes*, such as *Aminomonas* (2.21%), *Thermovirga* (3.00%) and *Cloacibacillus* (2.98%), also had higher abundance in the anaerobic sludge sample A-A. Sulfate-reducing bacteria *Desulfobacter* (6.94%), *Desulfomicrobium* (1.70%) and *Desulfobulbus* (0.96%) had higher abundance in the anaerobic sludge than in the aerobic samples, since sulfate is one common pollutant in tannery wastewater [29] and dissolved oxygen (DO) is limited in the UASB reactor.

In opposition, genera *Thauera*, Gp4 and *Caldilinea* dominated (>3%) in all the three aerobic samples, but had lower richness in the anaerobic sample A-A (abundance <0.60%) (Figure 2, Table S4). *Thauera* had extremely higher abundance in aerobic sludge samples B-D (12.95%), B-O (15.81%) and A-O (4.64%) than in anaerobic sludge sample A-A (0.11%). *Thauera* is known capable of denitrification and organic compounds biodegradation, which has been frequently detected in wastewater treatment bioreactors [30],[31]. *Caldilinea* (3.08–5.95%) consists of some filamentous species capable of flocs stabilization of activated sludge [32]. The genus Gp4 (4.20–16.74%) has not been well-described to date, but is known to dominate in the activated sludge of industrial [9] and municipal [18] WWTPs.

The functions of the four tannery activated sludge metagenomes were predicted by alignment of the Illumina sequencing reads against COG database in MG-RAST (Figure S3). Similar to the structural patterns of the microbial communities, the three aerobic sludge samples A-O, B-D and B-O showed similar functional profiles at the highest level of the COG category system (Figure S3). However, the anaerobic sludge showed different COG functional categories distribution, especially on the categories of ‘replication, recombination and repair’, ‘transcription’ and ‘translation, ribosomal structure and biogenesis’ (Figure S3). Table S5 shows that a total of nine identified COGs were responsible for denitrification (COG1140, COG2223, COG3005, COG3256, COG3420, COG4263 and COG5013) and nitrogen fixation (COG1348 and COG2710) in the aerobic or anaerobic sludge. Each category of the denitrifying functional genes had lower abundance in anaerobic sludge than in aerobic sludge. A previous study has also indicated that the abundance of denitrifying bacteria in an aerobic reactor was higher than in a nitrate-free anaerobic digester [33].

### Diversity of AOB and AOA amoA genes

**Figure 3:**
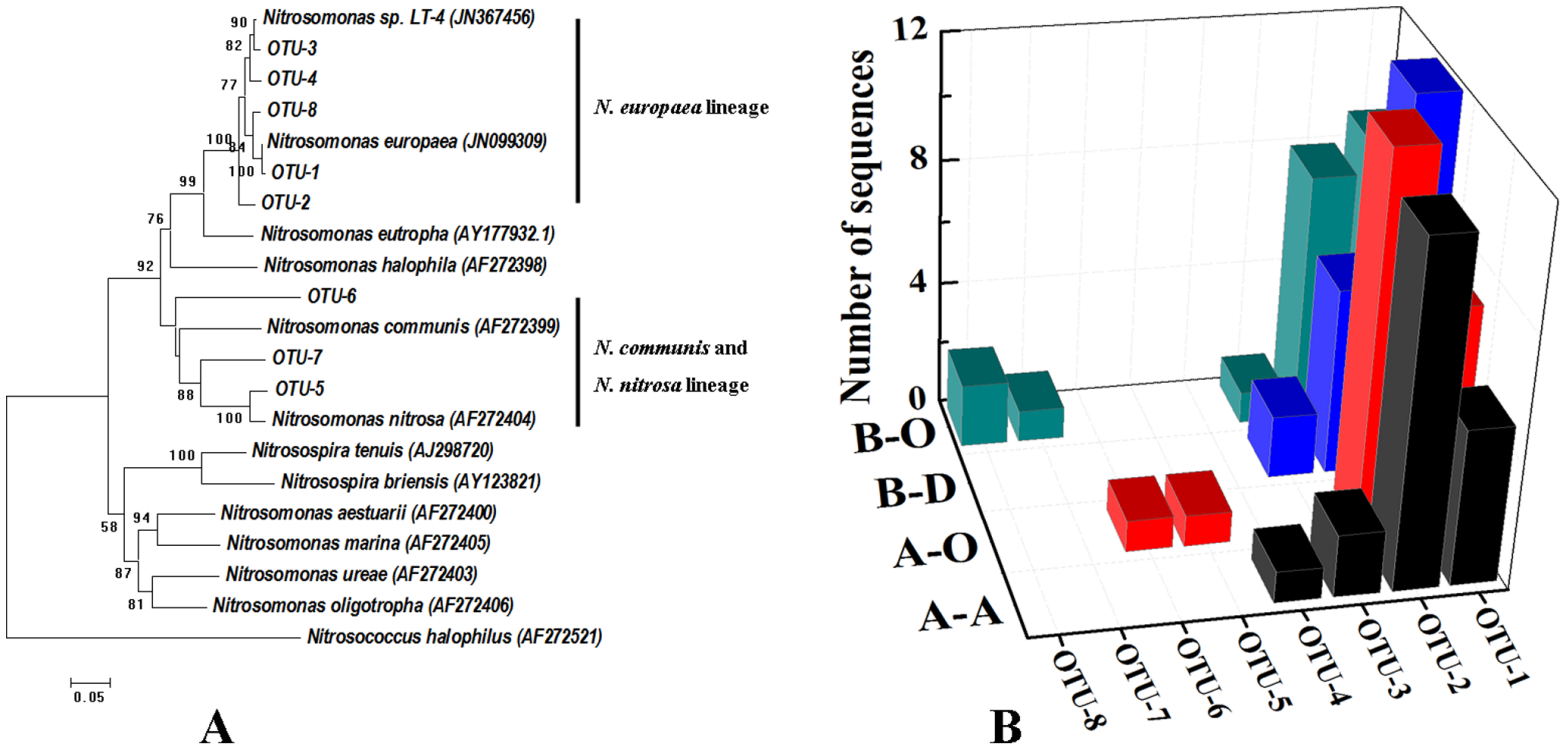
Neighbor-joining phylogenetic tree (A) and OTUs number (B) of AOB *amoA* gene in the four sludge samples. The evolutionary distances were computed using the Jukes–Cantor method. Bootstrap values (over 50) are indicated on branch nodes. Sequences obtained from this study are shown with “*OTU-*” in the names, and reference sequences were obtained from GenBank.

DNA cloning showed that a total of 8 OTUs were obtained at 3% distance cutoff from 81 AOB *amoA* gene sequences in 4 clone libraries (Figure 3A). The number of the OTUs recovered from individual libraries ranged from 3 (B-D) to 5 (B-O). OTU-1 and OTU-2 dominated in all samples, accounting for 86% of total sequences (Figure 3B). Phylogenetic analysis showed that all the bacterial *amoA* OTUs were affiliated to *Nitrosomonas europaea*, *Nitrosomonas communis* and *Nitrosomonas nitrosa* (Figure 3A), while no sequence in *Nitrosospira* lineage was observed. Previous studies have indicated that AOB *Nitrosomonas* instead of *Nitrosospira* dominates in WWTPs [3],[34]. Among the three species of *Nitrosomonas*, *N. europaea* dominated in all samples, and only a total of 3 sequences were affiliated to *N. communis* and *N. nitrosa* (Figure 3B). High concentration of ammonia (>170.0 mg/L) in the influent of the two WWTPs may contribute to the predominance of *N. europaea*, since previous studies have indicated that AOB of the *N. europaea* cluster are commonly found in the environments with high levels of ammonium [35]. In this study, similar AOB communities were observed in the UASB and the three aerobic bioreactors. Although DO is a limiting factor -of AOB growth in bioreactors, it has been indicated *N. europaea* lineage can enrich in both the high-DO and low-DO chemostats [36]. This is also supported by some previous studies revealing that *N. europaea* was the predominant AOB in various environments containing different levels of DO, such as laboratory-scale anaerobic ammonium-oxidizing reactors [37], full-scale modified Ludzack-Ettinger process WWTPs [38] and pilot-scale sequencing batch nitrifying reactors [39]. AOB in UASB may use the available oxygen to oxidize ammonia to nitrite, contributing to the anaerobic environments [40].

In the present study, we attempted to amplify AOA *amoA* gene from all the four samples by optimizing PCR conditions, but AOA *amoA* gene was detectable only in the anaerobic sludge sample A-A (Figure S4). Similarly, previous studies also revealed that AOA *amoA* is absent in aerobic sludge of different WWTPs [3],[41]. A total of 5 OTUs were obtained from 27 AOA *amoA* gene sequences in A-A clone library (Figure S5). It is known that most of environmental AOA are assigned to the clusters of Group I.1a (the marine and sediment lineage) and Group I.1b (the soil lineage) [42]. This study revealed that OTU-1 occupied 78% of the total AOA *amoA* gene sequences, and OTU-1, OTU-3 and OTU-4 had high similarity (>98%) to those AOA detected in sediment (clustered to the Group I.1a). Four sequences of OTU-2 and OTU-5 fell in the Group I.1b cluster. This result agrees with Mußmann et al. [41] indicating that all AOA *amoA* clones from tannery or refinery WWTPs were affiliated with Group I.1a.Ye and Zhang [43] also reported that AOA communities in a laboratory-scale bioreactor treating saline sewage were dominated by Group I.1a. However, the majority of AOA in many municipal WWTPs belonged to Group I.1b [3],[42]. The divergence may result from the difference in wastewater salinity, an important factor in shaping AOA communities [44].

### Abundance of amoA, nirK, nirS and nosZ genes

**Figure 4:**
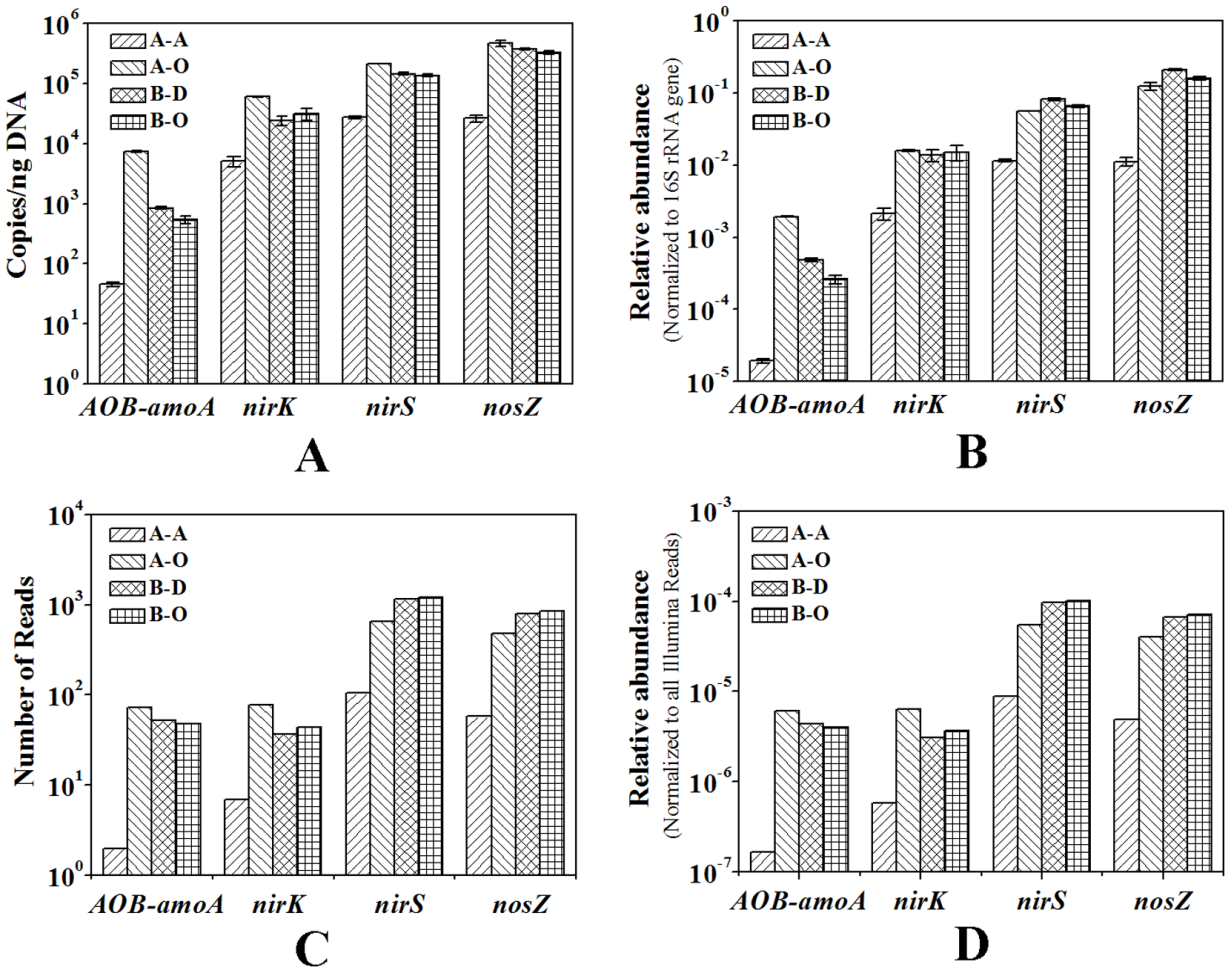
Abundance of *amoA*, *nirK*, *nirS* and *nosZ* genes in the four sludge samples revealed by qPCR (A and B) and metagenomic (alignment) approaches (C and D). A: relative abundance of the genes normalized to the mass of the extracted DNA; B: relative abundance of the genes normalized to the abundance of 16S rRNA gene; C: Number of the Illumina sequencing reads aligned to the genes sequences in the local database at identity cutoff>90% and alignment length>50 bp; D: Relative abundance of the genes in the Illumina dataset of each sludge sample.

In this study, the abundance of *amoA*, *nirK*, *nirS* and *nosZ* genes was quantified using both qPCR and metagenomic (alignment) approaches, and the later method has also been frequently used to quantify functional genes in the environment, including *amoA* gene in soil [8] and activated sludge [10]. Correlation analysis showed that the two methods generated consistent results (Figure S6). qPCR showed that the copy numbers of bacterial *amoA* gene in the four samples ranged from 45.8 to 7.33×10^3^ copies per ng DNA (Figure 4A). qPCR and metagenomic analysis consistently revealed that the relative abundance of bacterial *amoA* gene was higher in sample A-O than in the other sludge samples (Figure 4B and D). qPCR showed that the relative abundance of bacterial *amoA* gene to the overall bacterial population in the three aerobic sludge samples (0.03%–0.19%) was comparable to the previous reports of four membrane bioreactors (lower than 0.1%) capable of removing 70–95% of ammonium from different types of wastewater [45]. A previous study indicated that ammonia monooxygenase had high transcription activity in activated sludge [11]. Thus, the high ammonium removal efficiency in the two WWTPs may result from the high expression levels of ammonia monooxygenase and long hydraulic retention time (Table S1).

Nitrite reduction is usually catalyzed by two structurally different but functionally equivalent forms of nitrite reductase: copper and cytochrome *cd*_1_ -containing nitrite reductases encoded by the genes *nirK* and *nirS*, respectively. The copy numbers of *nirK* and *nirS* ranged from 5.05×10^3^ to 6.02×10^4^ and from 2.72×10^4^ to 2.14×10^5^ gene copies per ng DNA, respectively (Figure 4A). The average abundance of *nirS* and *nirK* relative to the overall bacterial population was 1.18% and 5.38%, respectively (Figure 4B). The relative abundance of *nirK* was found comparable to the results reported by Geets et al. (0.16–2.75%) [4]. qPCR and metagenomic analysis consistently indicated that A-O had higher abundance of *nirK* than the other samples. This study showed that *nirS* had higher abundance than *nirK* in each of the four samples (The ratio of *nirS/nirK* was over 3.0 in each sample). Similar results were observed in some WWTPs treating hospital wastewater, papermaking wastewater or pharmaceutical wastewater [4]. However, it cannot be indicated that *nirS* plays a greater role than *nirK* in the nitrogen removal, since expression level of the genes may affect their contribution to nitrogen removal [5]. Thus, further studies based on mRNA and protein expression have to be conducted.

N_2_O is an important component of greenhouse gas, and reduction of N_2_O to N_2_ in the environment has been attributed exclusively to the denitrifiers carrying *NosZ* gene. qPCR showed that the copy number of *nosZ* gene in the four sludge samples ranged from 2.63×10^4^ to 4.66×10^5^ gene copies per ng DNA (Figure 4A) with relative abundance within the range from 1.1% to 21.0% (Figure 4B). qPCR and metagenomic analysis consistently indicated that *nosZ* gene preferred aerobic environment (Figure 4). In anaerobic sludge, the abundance of *nosZ* gene were lower than the total abundance of *nirK* and *nirS*, indicating that some denitrifiers in the anaerobic sludge might lack *nosZ*, which may lead to the accumulation of N_2_O in anaerobic environments. Previous studies also indicated that low DO concentration in WWTPs favors N_2_O production during nitrification/denitrification process [46],[47], although nitrous oxide reductase is the most oxygen sensitive enzyme in the denitrification pathway [48].

It was found the effluent nitrate concentration has a significantly positive correlation with the relative abundance of the denitrifying genes *nirK* (R = 0.977, P = 0.023), *nirS* (R = 0.851, P = 0.149) and *nosZ* (R = 0.811, P = 0.189). This is supported by a previous study revealing significant correlation between nitrate concentration and the abundance of *nirK* or *nirS* in soil [5].

### Metagenomic analysis of nitrifying and denitrifying bacteria populations

16S rRNA gene pyroseqencing was conducted to investigate the abundance and diversity of AOB and NOB in the sludge samples. Results showed that *Nitrosomonas* and *Nitrosospira*, the two important genera of AOB in WWTPs [3], accounted for 0–0.34% and 0–0.20% of the total sequences of the samples, respectively (Table S6). Similarly, Zhang et al. [3] indicated that AOB occupied 0–0.64% of the total pyrosequencing sequences from six municipal WWTPs, and *Nitrosomonas* dominated in the WWTPs. Sample A-O had the highest abundance of AOB and NOB, followed by the other aerobic samples, and both AOB and NOB sequences were undetectable in the anaerobic sludge (A-A), which agrees with the qPCR results of *amoA* gene. A total of 76 AOB and 79 NOB sequences in all the samples were merged to construct phylogenetic tree to characterize the AOB and NOB communities, respectively. Similar to *amoA* gene diversity analysis, pyrosequencing showed that most of the AOB 16S rRNA sequences were grouped into *Nitrosomonas* sp., including *N. europaea* (5 OTUs), *N. ureae* (1 OTU) and *Nitrosomonas*-like species (3 OTUs) (Figure S7). Most of the *Nitrospira*-type NOB reads were grouped into *N. marina* (Figure S8), since tannery wastewater usually contains high concentration of salt (Table S1) [1].

Shapleigh [49] provided a list of 84 potential denitrifying bacterial genera, among which 17 genera were detectable in the four samples in this study (Table S7). The 11 types of potential denitrifying genera present in anaerobic sludge sample A-A accounted for 0.83% of the total pyrosequencing reads, which was lower than the corresponding proportions of the three aerobic sludge samples (8.22–20.52%). This agrees with the COG function alignment results, and lack of nitrate (Table S1) in UASB may be responsible for the low abundance of the denitrifiers.

Additionally, the Illumina reads annotated as *amoA*, *nirK*, *nirS* and *nosZ* genes were extracted and then assigned to specific host bacteria at genus level by BLASTX against NCBI-nr database and MEGAN. Unfortunately, most of the extracted reads had higher similarity to the corresponding genes of uncultured microorganisms. A total of 26 reads of *amoA*, 36 reads of *nirK*, 1,062 reads of *nirS* and 754 reads of *nosZ* in the samples were well assigned to specific host of 89 known genera (Figure S9). It should be noted that using the short Illumina sequencing reads in taxonomic assignment could produce unreliable results at low taxonomic levels [50], and the possible horizontal gene transfer of denitrifying genes among different taxa is likely to increase uncertainty of the results [51]. To maximize the reliability of the obtained results, only the highly abundant genera identified by both pyrosequencing and Illumina sequencing in this study were considered as the main nitrifiers or denitrifiers in the two tannery WWTPs.

All the *amoA* gene reads were assigned to *Nitrosomonas*, which agrees with *amoA* gene cloning results. Results showed that *Nitrosomonas* was the main host of nitrite reductase gene *nirK* in the samples A-A, B-D and B-O. This result is supported by Beaumont et al. [52] indicating presence of *nirK* in *Nitrosomonas europaea* cells. Interestingly, *nirK* in sample A-O was mainly carried by the bacterial cells of *Rhizobium*, not *Nitrosomonas* (Figure S9). This is consistent with the pyrosequencing results demonstrating that *Rhizobium* had high abundance in sample A-O (Table S4). *NirS* and *nosZ* genes were found mainly located in the bacterial cells of *Thauera*, *Paracoccus*, *Hyphomicrobium*, *Comamonas* and *Azoarcus* (Figure S9) dominating in the aerobic sludge (Table S4), revealing the crucial roles of the microorganisms in denitrification in aerobic environments. It is known that *Thaurea mechernichensis* [53] and *Paracoccus denitrificans* [54] can reduce nitrate even in the presence of high concentrations of O_2_.

In conclusion, combined use of DNA cloning, qPCR, 454 pyrosequencing and Illumina sequencing provided a comprehensive insight in microbial community structure of nitrifiers and denitrifiers in tannery WWTPs. *Proteobacteria* and *Synergistetes* phyla had highest abundance in aerobic and anaerobic sludge, respecitively. *Nitrosomonas europaea* dominated the ammonia-oxidizing community. *Thauera*, *Paracoccus*, *Hyphomicrobium*, *Comamonas* and *Azoarcus* were the predominant potential denitrifying genera, which may greatly contribute to the nitrogen removal in the WWTPs. qPCR and metagenomic approaches consistently revealed that functional genes *amoA*, *nirK*, *nirS* and *nosZ* had higher abundance in aerobic sludge than in anaerobic sludge. The results can extend our biological knowledge about nitrifiers and denitrifiers in tannery WWTPs, and might be practically useful in efficiently removing nitrogen from industrial wastewater.

## Supporting Information

Figure S1 **Rarefaction curves of 4 sludge samples at cutoff levels of 3% (solid lines) and 5% (dash lines).** The rarefaction curve, plotting the number of observed OTUs as a function of the number of sequences, was computed using RDP's pyrosequencing pipeline. The error bars show 95% confidence intervals.
(DOCX)

Figure S2 **Percentages of unclassified sequences at six taxonomic ranks for four sludge samples from two full-scale tannery wastewater treatment plants.** Effective bacterial sequences were classified using RDP Classifier at a confidence threshold of 50%.
(DOCX)

Figure S3 **Relative abundance of 21 COG functional categories in four tannery activated sludge metagenomes.** Relative abundances of functional categories were estimated by normalizing to the total number of protein coding sequences assigned to each corresponding functions. Designations of functional categories: A: Replication, recombination and repair, B: RNA processing and modification, C: Transcription, D: Translation, ribosomal structure and biogenesis, E: Cell cycle control, cell division, chromosome partitioning, F: Cell wall/membrane/envelope biogenesis, G: Cytoskeleton, H: Defense mechanisms, I: Intracellular trafficking, secretion, and vesicular transport, J: Posttranslational modification, protein turnover, chaperones, K: Signal transduction mechanisms, L: Amino acid transport and metabolism, M: Carbohydrate transport and metabolism, N: Coenzyme transport and metabolism, O: Energy production and conversion, P: Inorganic ion transport and metabolism, Q: Lipid transport and metabolism, R: Nucleotide transport and metabolism, S: Secondary metabolites biosynthesis, transport and catabolism, T: Function unknown, U: General function prediction only.
(DOCX)

Figure S4 **Gel image of PCR products of AOA** ***amoA*** **gene (M: DL2000 DNA Marker; 0: Negative control; 1-2: A-A; 3-4: A-O; 5-6: B-D; 7-8: B-O).**
(DOCX)

Figure S5 **Neighbor-joining phylogenetic tree of AOA** ***amoA*** **gene sequences.** The evolutionary distances were computed using the Jukes–Cantor method. Bootstrap values (over 50) are indicated on branch nodes.
(DOCX)

Figure S6 **Correlation between the qPCR and metagenomic (alignment) approaches for the quantification of AOB** ***amoA*** **,** ***nirK*** **,** ***nirS*** **and** ***nosZ*** **genes.** The abundance of the four genes obtained by qPCR were normalized to the total bacterial community, and the metagenomic alignment results were normalized to the total number of clean Illumina sequencing reads in each sample.
(DOCX)

Figure S7 **Neighbor-joining phylogenetic tree of AOB 16S rRNA gene sequences.** The evolutionary distances were computed using the Jukes–Cantor method. Bootstrap values are indicated on branch nodes. Sequences obtained from 454-pyrosequencing in this study are shown with “OTU-” in the names, and the number in “| | | | ” represented the number of AOB 16S rRNA gene sequences in the samples A-O, B-D and B-O, respectively. The information of reference sequences was obtained from GenBank.
(DOCX)

Figure S8 **Neighbor-joining phylogenetic tree of NOB 16S rRNA gene sequences.** The evolutionary distances were computed using the Jukes–Cantor method. Bootstrap values are indicated on branch nodes. Sequences obtained from 454-pyrosequencing in this study are shown with “OTU-” in the names, and the number in “| | | |” represented the number of AOB 16S rRNA gene sequences in sample A-O, B-D and B-O, respectively. The information of reference sequences was obtained from GenBank.
(DOCX)

Figure S9 **Functional microorganisms in nitrification and denitrification processes.** Based on the BLASTX against NCBI-nr database and MEGAN, the Illumina clean reads annotated as *amoA*, *nirK*, *nirS* or *nosZ* genes were assigned to specific genera, and 59 genera of bacteria with more than two hits in at least one sample are displayed in the heat map which was generated using R (version 3.01).
(DOCX)

Table S1 **Operational parameters of the two tannery wastewater treatment plants and concentrations of metallic elements in the influent wastewater.**
(DOCX)

Table S2 **Primers used for PCR and qPCR in this study.**
(DOCX)

Table S3 **Information of 16S rRNA gene pyrosequencing reads and biodiversity of the four activated sludge samples from two tannery wastewater treatment plants.**
(DOCX)

Table S4 **Abundance of all genera in the four sludge samples.** The abundance is presented in terms of percentages of the total sequences (6471 sequences) in a sample. Sorted alphabetically by phylum and genus. The bold numbers indicate the genera with relative abundance>1%.
(DOCX)

Table S5 **COGs related to denitrification and nitrogen fixation identified in the four activated sludge metagenomes.**
(DOCX)

Table S6 **Composition of nitrifying bacteria (AOB and NOB) in the four sludge samples revealed by 454 pyrosequencing.**
(DOCX)

Table S7 **Potential denitrifying genera detected in the four sludge samples through 454 pyrosequencing.** The reads number was calculated by normalizing the total pyrosequencing reads to 6,471 for each sample.
(DOCX)
