# Supplementary material for: Abundance and Diversity of Bacterial Nitrifiers and Denitrifiers and Their Functional Genes in Tannery Wastewater Treatment Plants Revealed by High-Throughput Sequencing
Source: PLoS One. 2014 Nov 24;9(11):e113603. doi: 10.1371/journal.pone.0113603 (PMC4242629; doi:10.1371/journal.pone.0113603)
Supplement: Figure S1 — Rarefaction curves of 4 sludge samples at cutoff levels of 3% (solid lines) and 5% (dash lines). The rarefaction curve, plotting the number of observed OTUs as a function of the number of sequences, was computed using RDP's pyrosequencing pipeline. The error bars show 95% confidence intervals. (DOCX) [file pone.0113603.s001.docx]

**Figure S1** **Rarefaction curves of the four sludge samples at cutoff levels of 3% (*solid lines*) and 5% (*dash lines*).** The rarefaction curve, plotting the number of observed OTUs as a function of the number of sequences, was computed using RDP’s pyrosequencing pipeline. The error bars show 95% confidence intervals.

**
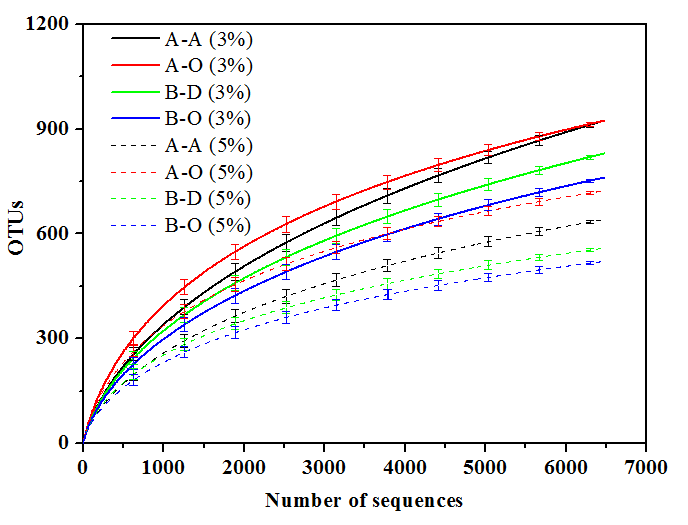
**
