# Supplementary material for: Abundance and Diversity of Bacterial Nitrifiers and Denitrifiers and Their Functional Genes in Tannery Wastewater Treatment Plants Revealed by High-Throughput Sequencing
Source: PLoS One. 2014 Nov 24;9(11):e113603. doi: 10.1371/journal.pone.0113603 (PMC4242629; doi:10.1371/journal.pone.0113603)
Supplement: Figure S2 — Percentages of unclassified sequences at six taxonomic ranks for four sludge samples from two full-scale tannery wastewater treatment plants. Effective bacterial sequences were classified using RDP Classifier at a confidence threshold of 50%. (DOCX) [file pone.0113603.s002.docx]

**Figure S2** **Percentages of unclassified sequences at six taxonomic ranks for the four sludge samples from two full-scale tannery wastewater treatment plants.** Effective bacterial sequences were classified using RDP Classifier at a confidence threshold of 50%.

**
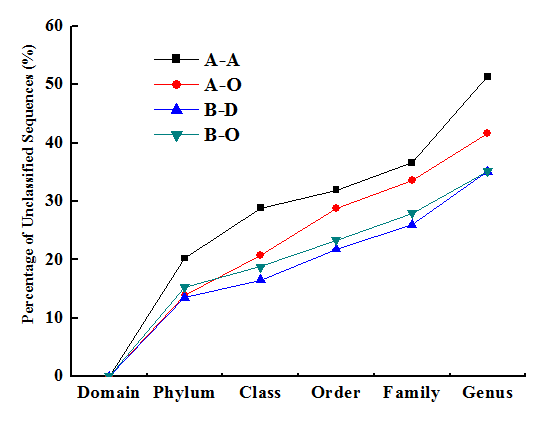
**
