# Supplementary material for: Abundance and Diversity of Bacterial Nitrifiers and Denitrifiers and Their Functional Genes in Tannery Wastewater Treatment Plants Revealed by High-Throughput Sequencing
Source: PLoS One. 2014 Nov 24;9(11):e113603. doi: 10.1371/journal.pone.0113603 (PMC4242629; doi:10.1371/journal.pone.0113603)
Supplement: Figure S3 — Relative abundance of 21 COG functional categories in four tannery activated sludge metagenomes. Relative abundances of functional categories were estimated by normalizing to the total number of protein coding sequences assigned to each corresponding functions. Designations of functional categories: A: Replication, recombination and repair, B: RNA processing and modification, C: Transcription, D: Translation, ribosomal structure and biogenesis, E: Cell cycle control, cell division, chromosome partitioning, F: Cell wall/membrane/envelope biogenesis, G: Cytoskeleton, H: Defense mechanisms, I: Intracellular trafficking, secretion, and vesicular transport, J: Posttranslational modification, protein turnover, chaperones, K: Signal transduction mechanisms, L: Amino acid transport and metabolism, M: Carbohydrate transport and metabolism, N: Coenzyme transport and metabolism, O: Energy production and conversion, P: Inorganic ion transport and metabolism, Q: Lipid transport and metabolism, R: Nucleotide transport and metabolism, S: Secondary metabolites biosynthesis, transport and catabolism, T: Function unknown, U: General function prediction only. (DOCX) [file pone.0113603.s003.docx]

**Figure S3 Relative abundance of 21 COG functional categories in four tannery activated sludge metagenomes.** Relative abundances of functional categories were estimated by normalizing to the total number of protein coding sequences assigned to each corresponding function.


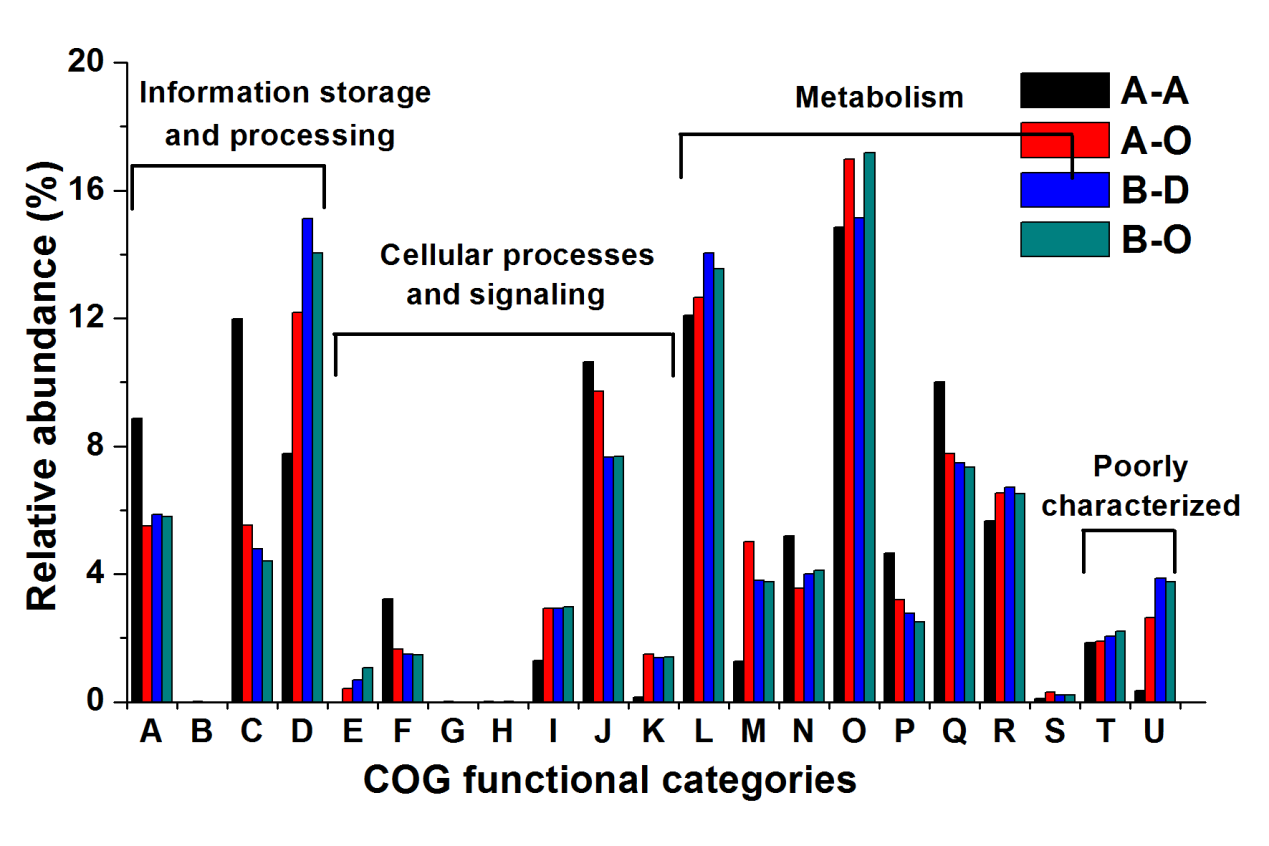


Designations of functional categories: A: Replication, recombination and repair, B: RNA processing and modification, C: Transcription, D: Translation, ribosomal structure and biogenesis, E: Cell cycle control, cell division, chromosome partitioning, F: Cell wall/membrane/envelope biogenesis, G: Cytoskeleton, H: Defense mechanisms, I: Intracellular trafficking, secretion, and vesicular transport, J: Posttranslational modification, protein turnover, chaperones, K: Signal transduction mechanisms, L: Amino acid transport and metabolism, M: Carbohydrate transport and metabolism, N: Coenzyme transport and metabolism, O: Energy production and conversion, P: Inorganic ion transport and metabolism, Q: Lipid transport and metabolism, R: Nucleotide transport and metabolism, S: Secondary metabolites biosynthesis, transport and catabolism, T: Function unknown, U: General function prediction only.
