# Supplementary material for: Abundance and Diversity of Bacterial Nitrifiers and Denitrifiers and Their Functional Genes in Tannery Wastewater Treatment Plants Revealed by High-Throughput Sequencing
Source: PLoS One. 2014 Nov 24;9(11):e113603. doi: 10.1371/journal.pone.0113603 (PMC4242629; doi:10.1371/journal.pone.0113603)
Supplement: Figure S4 — Gel image of PCR products of AOA amoA gene (M: DL2000 DNA Marker; 0: Negative control; 1-2: A-A; 3-4: A-O; 5-6: B-D; 7-8: B-O). (DOCX) [file pone.0113603.s004.docx]

**Figure S4 Gel image of PCR products of AOA *amoA* gene** (M: DL2000 DNA Marker; 0: Negative control; 1-2: A-A; 3-4: A-O; 5-6: B-D; 7-8: B-O).

**

**
