# Supplementary material for: Abundance and Diversity of Bacterial Nitrifiers and Denitrifiers and Their Functional Genes in Tannery Wastewater Treatment Plants Revealed by High-Throughput Sequencing
Source: PLoS One. 2014 Nov 24;9(11):e113603. doi: 10.1371/journal.pone.0113603 (PMC4242629; doi:10.1371/journal.pone.0113603)

**Figure S5 Neighbor-joining phylogenetic tree based on AOA *amoA* gene sequences.** The evolutionary distances were computed using the Jukes–Cantor method. Bootstrap values (over 50) are indicated on branch nodes.


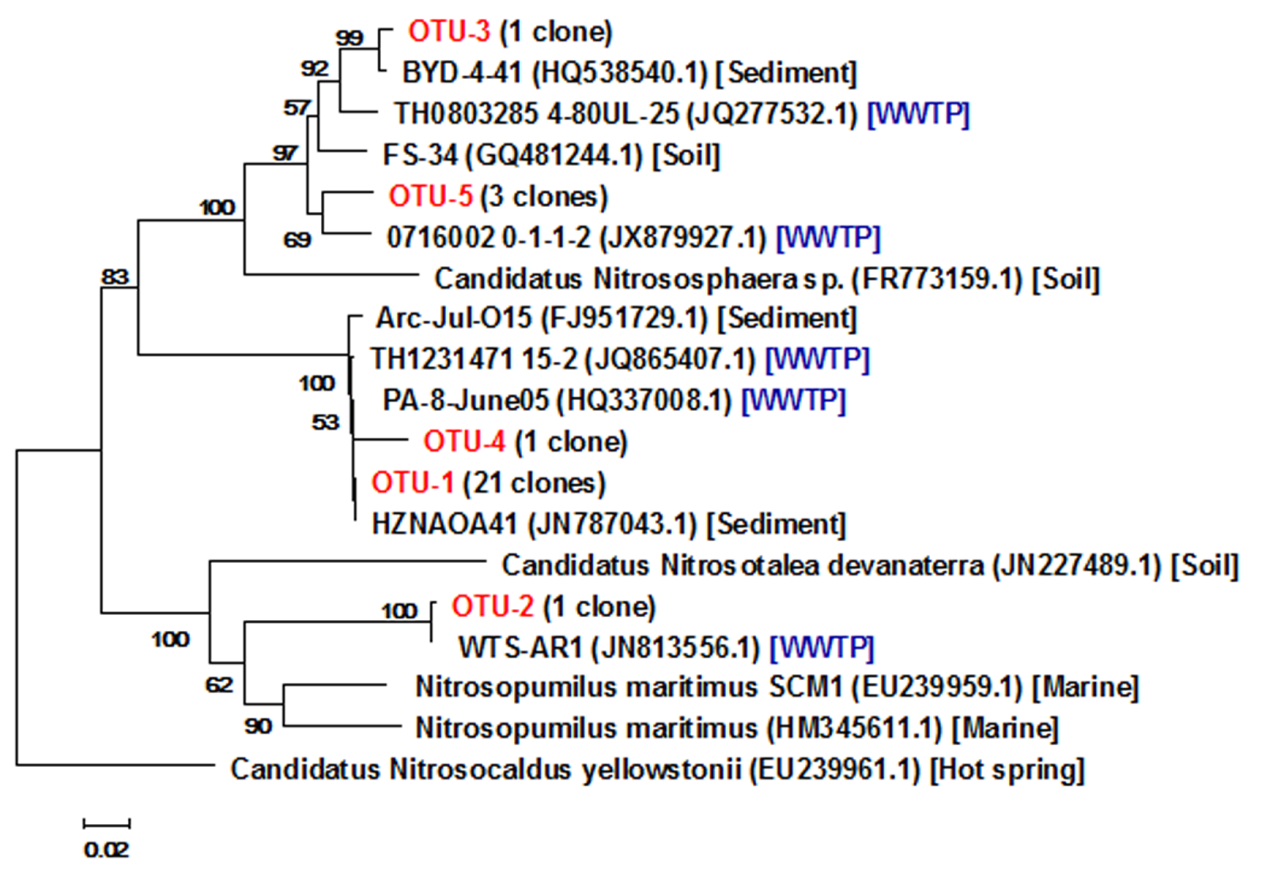

Supplement: Figure S5 — Neighbor-joining phylogenetic tree of AOA amoA gene sequences. The evolutionary distances were computed using the Jukes–Cantor method. Bootstrap values (over 50) are indicated on branch nodes. (DOCX) [file pone.0113603.s005.docx]
