# Supplementary material for: Abundance and Diversity of Bacterial Nitrifiers and Denitrifiers and Their Functional Genes in Tannery Wastewater Treatment Plants Revealed by High-Throughput Sequencing
Source: PLoS One. 2014 Nov 24;9(11):e113603. doi: 10.1371/journal.pone.0113603 (PMC4242629; doi:10.1371/journal.pone.0113603)

**Figure S6 Correlation between the qPCR and metagenomic (alignment) approaches for the quantification of AOB *amoA*, *nirK*, *nirS* and *nosZ* genes.** The abundance of the four genes obtained by qPCR were normalized to the total bacterial community, and the metagenomic alignment results were normalized to the total number of clean Illumina sequencing reads in each sample.


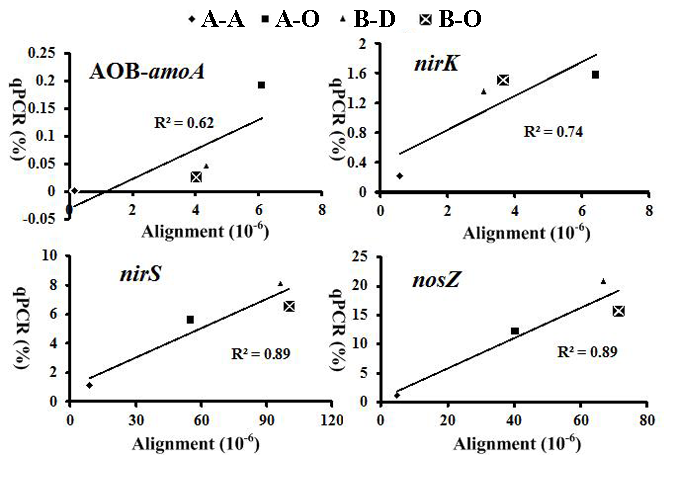

Supplement: Figure S6 — Correlation between the qPCR and metagenomic (alignment) approaches for the quantification of AOB amoA , nirK , nirS and nosZ genes. The abundance of the four genes obtained by qPCR were normalized to the total bacterial community, and the metagenomic alignment results were normalized to the total number of clean Illumina sequencing reads in each sample. (DOCX) [file pone.0113603.s006.docx]
