# Supplementary material for: Abundance and Diversity of Bacterial Nitrifiers and Denitrifiers and Their Functional Genes in Tannery Wastewater Treatment Plants Revealed by High-Throughput Sequencing
Source: PLoS One. 2014 Nov 24;9(11):e113603. doi: 10.1371/journal.pone.0113603 (PMC4242629; doi:10.1371/journal.pone.0113603)
Supplement: Figure S8 — Neighbor-joining phylogenetic tree of NOB 16S rRNA gene sequences. The evolutionary distances were computed using the Jukes–Cantor method. Bootstrap values are indicated on branch nodes. Sequences obtained from 454-pyrosequencing in this study are shown with “OTU-” in the names, and the number in “| | | |” represented the number of AOB 16S rRNA gene sequences in sample A-O, B-D and B-O, respectively. The information of reference sequences was obtained from GenBank. (DOCX) [file pone.0113603.s008.docx]

**Figure S8 Neighbor-joining phylogenetic tree based on NOB 16S rRNA gene sequences.** The evolutionary distances were computed using the Jukes–Cantor method. Bootstrap values are indicated on branch nodes. Sequences obtained from 454-pyrosequencing in this study are shown with “OTU-” in the names, and the number in “| | | |” represented the number of AOB 16S rRNA gene sequences in sample A-O, B-D and B-O, respectively. The information of reference sequences was obtained from GenBank.

**
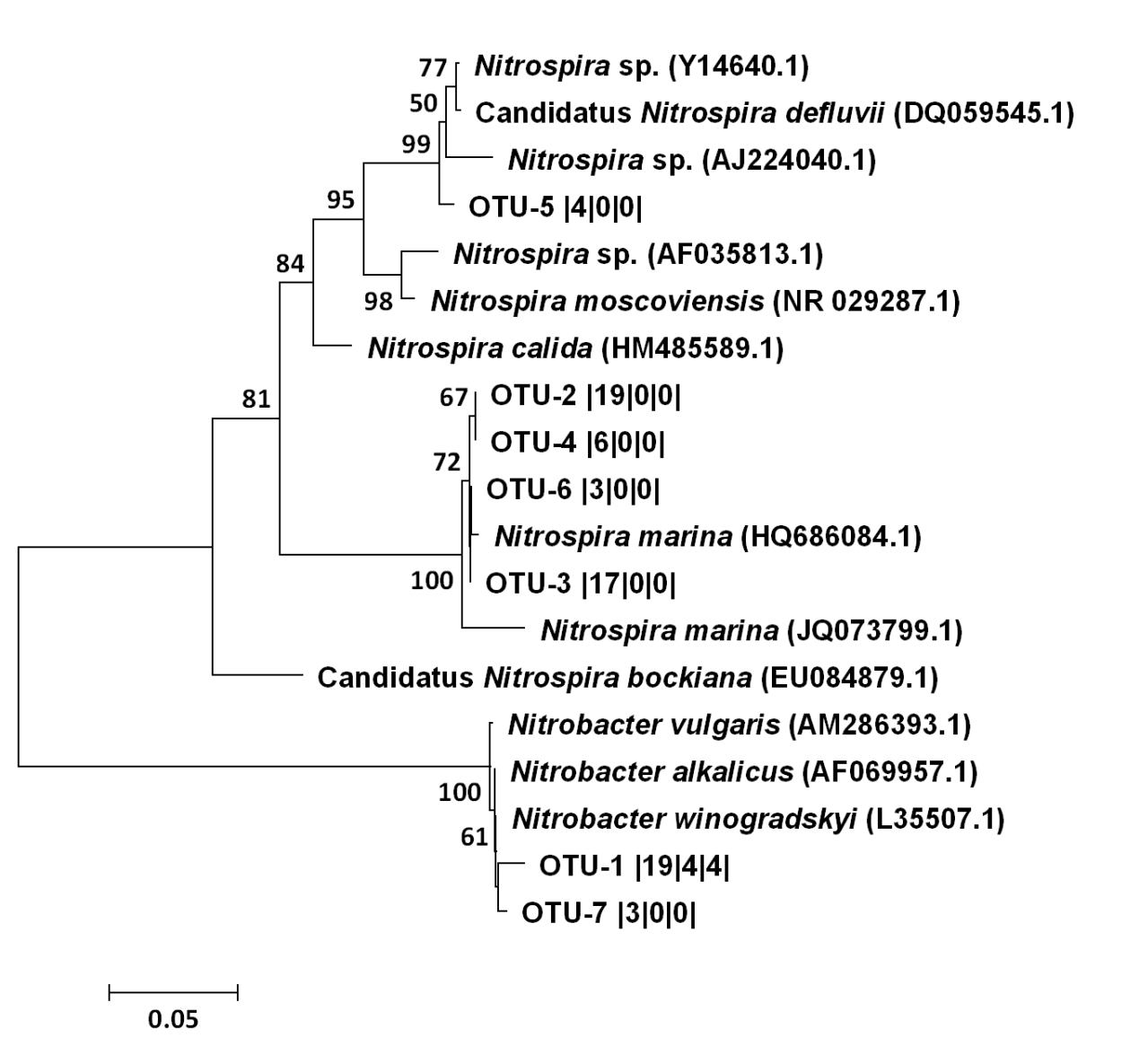
**
