# Supplementary material for: Abundance and Diversity of Bacterial Nitrifiers and Denitrifiers and Their Functional Genes in Tannery Wastewater Treatment Plants Revealed by High-Throughput Sequencing
Source: PLoS One. 2014 Nov 24;9(11):e113603. doi: 10.1371/journal.pone.0113603 (PMC4242629; doi:10.1371/journal.pone.0113603)

**Figure S9 Functional microorganisms in nitrification and denitrification processes.** Based on the BLASTX against NCBI-nr database and MEGAN, the Illumina clean reads annotated as *amoA*, *nirK*, *nirS* or *nosZ* genes were assigned to specific genera, and 59 genera of bacteria with more than two hits in at least one sample are displayed in the heat map which was generated using R (version 3.01).


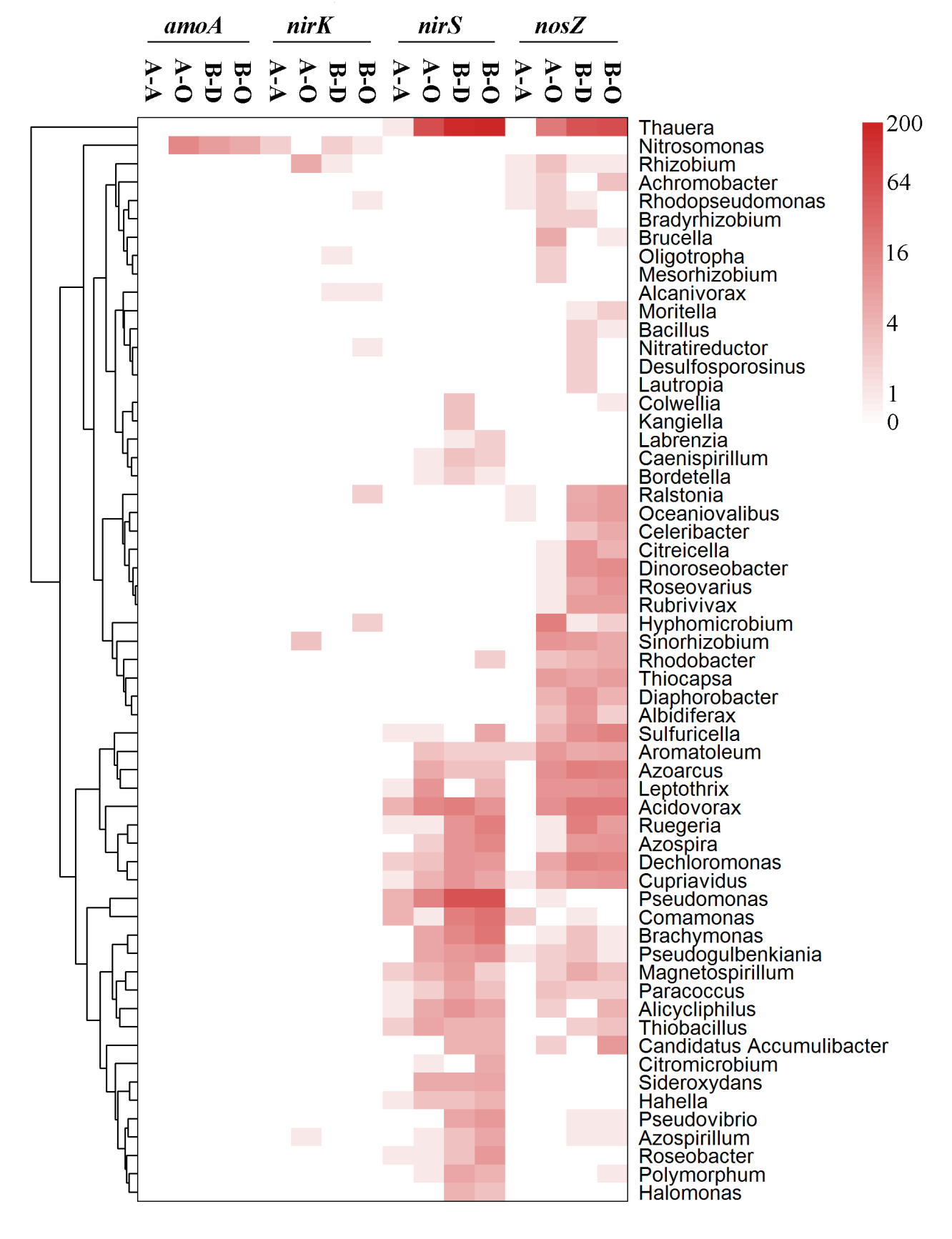

Supplement: Figure S9 — Functional microorganisms in nitrification and denitrification processes. Based on the BLASTX against NCBI-nr database and MEGAN, the Illumina clean reads annotated as amoA, nirK, nirS or nosZ genes were assigned to specific genera, and 59 genera of bacteria with more than two hits in at least one sample are displayed in the heat map which was generated using R (version 3.01). (DOCX) [file pone.0113603.s009.docx]
