# Supplementary material for: Abundance and Diversity of Bacterial Nitrifiers and Denitrifiers and Their Functional Genes in Tannery Wastewater Treatment Plants Revealed by High-Throughput Sequencing
Source: PLoS One. 2014 Nov 24;9(11):e113603. doi: 10.1371/journal.pone.0113603 (PMC4242629; doi:10.1371/journal.pone.0113603)
Supplement: Table S1 — Operational parameters of the two tannery wastewater treatment plants and concentrations of metallic elements in the influent wastewater. (DOCX) [file pone.0113603.s010.docx]

**Table S1 Operational parameters of the two tannery wastewater treatment plants and concentrations of metallic elements (mg/L)**

**in the influent wastewater.**

| Operational parameters | | | | | | | | | | | | |
| --- | --- | --- | --- | --- | --- | --- | --- | --- | --- | --- | --- | --- |
| WWTP | Code | Process | Flow rate (m^3^d^-1^) | HRT (h) | Influent (mg/L) | | | | Effluent (mg/L) | | | |
|  |  |  |  |  | COD | NH_4_^+^ | NO_2_^-^ | NO_3_^-^ | COD | NH_4_^+^ | NO_2_^-^ | NO_3_^-^ |
| WWTPA | A-A | UASB | 1,200 | 24 | 1332 | 177.4 | 0.1 | 2.0 | 610 | 181.6 | 0.04 | 1.3 |
|  | A-O | A/O | 1,200 | 48 | 610 | 181.6 | 0.04 | 1.3 | 78 | 5 | 0.7 | 77.3 |
| WWTPB | B-D | OD | 20,000 | 24 | 2066 | 210.7 | 0.05 | 2.2 | 585 | 125.8 | 0.6 | 65.6 |
|  | B-O | A/O | 20,000 | 48 | 585 | 125.8 | 0.6 | 65.6 | 80 | 4.6 | 0.8 | 91.5 |
| Concentrations of metallic elements (mg/L) in the influent wastewater | | | | | | | | | |  |  |  |
| Component | Na | Ca | K | Mg | Mn | Cr | Fe | Ba | Zn |  |  |  |
| WWTPA | 609.75 | 86.88 | 7.68 | 23.35 | 0.02 | 0.06 | 0.03 | 0.05 | 0.01 |  |  |  |
| WWTPB | 2775.50 | 85.55 | 38.75 | 81.60 | 0.36 | 0.01 | 1.52 | 0.06 | 0.04 |  |  |  |

Note: UASB, upflow anaerobic sludge blanket; A/O, anoxic/aerobic; OD, oxidation ditch; HRT, hydraulic retention time.
