# Supplementary material for: Abundance and Diversity of Bacterial Nitrifiers and Denitrifiers and Their Functional Genes in Tannery Wastewater Treatment Plants Revealed by High-Throughput Sequencing
Source: PLoS One. 2014 Nov 24;9(11):e113603. doi: 10.1371/journal.pone.0113603 (PMC4242629; doi:10.1371/journal.pone.0113603)
Supplement: Table S2 — Primers used for PCR and qPCR in this study. (DOCX) [file pone.0113603.s011.docx]

**Table S2 Primers used for PCR and qPCR in this study.**

| Target | primers | Sequence (5’-3’) | Fragment  Size  (bp) | Annealing  Temp (^0^C) | Reference |
| --- | --- | --- | --- | --- | --- |
| AOA-*amoA* | Arch-amoAF  Arch-amoAR | STAATGGTCTGGCTTAGACG  GCGGCCATCCATCTGTATGT | 635 | 53 | [1]  [1] |
| AOB-*amoA* | amoA-1F  amoA-2R | GGGGTTTCTACTGGTGGT  CCCCTCGGCAAAGCCTTCTTC | 491 | 56 | [2]  [2] |
| *nirS* | cd3aF  R3cd | GTSAACGTSAAGGARACSGG  GASTTCGGRTGSGTCTTGA | 425 | 59 | [3]  [4] |
| *nirK* | F1aCu  R3Cu | ATCATGGTSCTGCCGCG  GCCTCGATCAGRTTGTGGTT | 473 | 59 | [5]  [5] |
| *nosZ* | nosZ2F  nosZ2R | CGCRACGGCAASAAGGTSMSSGT  CAKRTGCAKSGCRTGGCAGAA | 267 | 59 | [6]  [6] |
| Bacterial  16S rRNA | 341f  515r | CCTACGGGAGGCAGCAG  AATCCGCGGCTGGCA | 174 | 60 | [7]  [7] |

**References**

1. Francis, C.A., Roberts, K.J., Beman, J.M., Santoro, A.E., Oakley, B.B., 2005. Ubiquity and diversity of ammonia-oxidizing archaea in water columns and sediments of the ocean. Proc. Natl. Acad. Sci. U.S.A. 102, 14683-14688.
2. Rotthauwe, J.H., Witzel, K.P., Liesack, W., 1997. The ammonia monooxygenase structural gene *amoA* as a functional marker: molecular fine-scale analysis of natural ammonia-oxidizing populations. Appl. Environ. Microbiol. 63, 4704-4712.
3. Michotey, V., Mejean, V., Bonin, P., 2000. Comparison of methods for quantification of cytochrome cd(1)-denitrifying bacteria in environmental marine samples. Appl. Environ. Microbiol. 66, 1564-1571.
4. Throback, I.N., Enwall, K., Jarvis, A., Hallin, S., 2004. Reassessing PCR primers targeting *nirS*, *nirK* and *nosZ* genes for community surveys of denitrifying bacteria with DGGE. FEMS Microbiol. Ecol. 49, 401-417.
5. Hallin, S., Lindgren, P.E., 1999. PCR detection of genes encoding nitrite reductase in denitrifying bacteria. Appl. Environ. Microbiol. 65, 1652-1657.
6. Henry, S., Bru, D., Stres, B., Hallet, S., Philippot, L., 2006. Quantitative detection of the *nosZ* gene, encoding nitrous oxide reductase, and comparison of the abundances of 16S rRNA, *narG*, *nirK*, and *nosZ* genes in soils. Appl. Environ. Microbiol. 72, 5181-5189.
7. Lopez-Gutierrez, J.C., Henry, S., Hallet, S., Martin-Laurent, F., Catroux, G., Philippot, L., 2004. Quantification of a novel group of nitrate-reducing bacteria in the environment by real-time PCR. J. Microbiol. Methods 57, 399-407.
