# Supplementary material for: Abundance and Diversity of Bacterial Nitrifiers and Denitrifiers and Their Functional Genes in Tannery Wastewater Treatment Plants Revealed by High-Throughput Sequencing
Source: PLoS One. 2014 Nov 24;9(11):e113603. doi: 10.1371/journal.pone.0113603 (PMC4242629; doi:10.1371/journal.pone.0113603)
Supplement: Table S3 — Information of 16S rRNA gene pyrosequencing reads and biodiversity of the four activated sludge samples from two tannery wastewater treatment plants. (DOCX) [file pone.0113603.s012.docx]

**Table S3** **Information of 16S rRNA gene pyrosequencing reads and biodiversity of the four activated sludge samples from two tannery wastewater treatment plants.**

| Sample code | Read | | | | | 3% cutoff | | | 5% cutoff | | |
| --- | --- | --- | --- | --- | --- | --- | --- | --- | --- | --- | --- |
|  | Raw | Denoised | Effective  (chimera removed) | Archaeal | Bacterial | OTUs | Shannon | GOOD's coverage (%) | OTUs | Shannon | GOOD's coverage (%) |
| A-A | 19150 | 9337 | 9311 | 37 | 9274 | 923 | 5.5 | 93.2% | 641 | 4.9 | 96.0% |
| A-O | 23554 | 13790 | 13730 | 3 | 13727 | 923 | 5.9 | 94.7% | 723 | 5.6 | 96.4% |
| B-D | 13126 | 8068 | 8050 | 1 | 8049 | 829 | 5.3 | 94.3% | 559 | 4.9 | 96.9% |
| B-O | 11506 | 6491 | 6471 | 0 | 6471 | 760 | 5.2 | 95.0% | 521 | 4.7 | 97.1% |

**Note:** OTUs: operational taxonomic units; Shannon: Shannon's diversity index; The biodiversity indices (OTUs, Shannon and GOOD’s coverage) were calculated by normalizing the number of the filtered sequences from each sludge sample to 6471.
