# Supplementary material for: Abundance and Diversity of Bacterial Nitrifiers and Denitrifiers and Their Functional Genes in Tannery Wastewater Treatment Plants Revealed by High-Throughput Sequencing
Source: PLoS One. 2014 Nov 24;9(11):e113603. doi: 10.1371/journal.pone.0113603 (PMC4242629; doi:10.1371/journal.pone.0113603)
Supplement: Table S4 — Abundance of all genera in the four sludge samples. The abundance is presented in terms of percentages of the total sequences (6471 sequences) in a sample. Sorted alphabetically by phylum and genus. The bold numbers indicate the genera with relative abundance>1%. (DOCX) [file pone.0113603.s013.docx]

**Table S4 Abundance of all genera in the four sludge samples.** The abundance is presented in terms of percentages of the total sequences (6471 sequences) in a sample. Sorted alphabetically by phylum and genus. The bold numbers indicate the genera with relative abundance >1%.

| Phylum | Genus | A-A | A-O | B-D | B-O |
| --- | --- | --- | --- | --- | --- |
| *Acidobacteria* | *Gp10* | 0.00 | 0.02 | 0.00 | 0.02 |
|  | *Gp16* | 0.59 | **5.42** | 0.26 | 0.36 |
|  | *Gp17* | 0.09 | 0.03 | 0.00 | 0.00 |
|  | *Gp18* | 0.17 | 0.00 | 0.00 | 0.02 |
|  | *Gp21* | 0.06 | 0.00 | 0.00 | 0.00 |
|  | *Gp3* | 0.00 | 0.29 | 0.19 | 0.09 |
|  | *Gp4* | 0.06 | **4.20** | **14.54** | **16.74** |
|  | *Gp6* | 0.43 | **3.32** | 0.82 | 0.91 |
|  | *Gp7* | 0.00 | 0.02 | 0.00 | 0.00 |
| *Actinobacteria* | *Aciditerrimonas* | 0.02 | 0.25 | 0.15 | 0.00 |
|  | *Brevibacterium* | 0.00 | 0.02 | 0.00 | 0.00 |
|  | *Conexibacter* | 0.03 | 0.68 | 0.00 | 0.02 |
|  | *Corynebacterium* | 0.00 | 0.05 | 0.00 | 0.02 |
|  | *Cryobacterium* | 0.00 | 0.00 | 0.08 | 0.03 |
|  | *Dietzia* | 0.00 | 0.02 | 0.00 | 0.00 |
|  | *Ferrimicrobium* | 0.00 | 0.02 | 0.00 | 0.00 |
|  | *Iamia* | 0.00 | 0.08 | 0.02 | 0.00 |
|  | *Ilumatobacter* | 0.00 | 0.32 | 0.19 | 0.17 |
|  | *Kineosphaera* | 0.00 | 0.00 | 0.02 | 0.00 |
|  | *Leifsonia* | 0.00 | 0.00 | 0.02 | 0.00 |
|  | *Leucobacter* | 0.00 | 0.36 | 0.05 | 0.02 |
|  | *Marmoricola* | 0.00 | 0.12 | 0.02 | 0.00 |
|  | *Mobilicoccus* | 0.00 | 0.03 | 0.00 | 0.00 |
|  | *Mycobacterium* | 0.02 | 0.20 | 0.05 | 0.11 |
|  | *Nocardioides* | 0.00 | 0.23 | 0.03 | 0.02 |
|  | *Olsenella* | 0.02 | 0.00 | 0.00 | 0.00 |
|  | *Ornithinimicrobium* | 0.00 | 0.00 | 0.00 | 0.02 |
|  | *Pimelobacter* | 0.00 | 0.02 | 0.00 | 0.00 |
|  | *Piscicoccus* | 0.00 | 0.00 | 0.05 | 0.00 |
|  | *Pseudonocardia* | 0.00 | 0.05 | 0.00 | 0.00 |
|  | *Salinibacterium* | 0.00 | 0.00 | 0.02 | 0.00 |
|  | *Solirubrobacter* | 0.00 | 0.03 | 0.00 | 0.00 |
|  | *Thermoleophilum* | 0.06 | 0.11 | 0.03 | 0.05 |
|  | *Yaniella* | 0.02 | 0.00 | 0.00 | 0.00 |
| *Armatimonadetes* | *Armatimonadetes_gp5* | 0.00 | 0.02 | 0.00 | 0.00 |
| *Bacteroidetes* | *Aequorivita* | 0.00 | 0.00 | 0.23 | 0.28 |
|  | *Alistipes* | 0.00 | 0.00 | 0.15 | 0.00 |
|  | *Alkaliflexus* | 0.02 | 0.00 | 0.00 | 0.00 |
|  | *Arenibacter* | 0.05 | 0.00 | 0.00 | 0.00 |
|  | *Bacteroides* | 0.02 | 0.05 | 0.49 | 0.00 |
|  | *Barnesiella* | 0.02 | 0.00 | **2.75** | 0.02 |
|  | *Bizionia* | 0.00 | 0.00 | 0.02 | 0.00 |
|  | *Cellulophaga* | 0.20 | 0.00 | 0.00 | 0.00 |
|  | *Ferruginibacter* | 0.03 | 0.23 | 0.02 | 0.11 |
|  | *Flavihumibacter* | 0.02 | 0.00 | 0.00 | 0.02 |
|  | *Flavitalea* | 0.00 | 0.02 | 0.00 | 0.00 |
|  | *Flavobacterium* | 0.02 | 0.02 | 0.06 | 0.00 |
|  | *Fluviicola* | 0.00 | 0.09 | 0.00 | 0.00 |
|  | *Fontibacter* | 0.02 | 0.00 | 0.00 | 0.00 |
|  | *Fulvivirga* | 0.00 | 0.05 | 0.32 | 0.28 |
|  | *Gracilimonas* | 0.00 | 0.00 | 0.02 | 0.00 |
|  | *Haliscomenobacter* | 0.00 | 0.09 | 0.00 | 0.03 |
|  | *Lewinella* | 0.00 | 0.15 | 0.25 | 0.28 |
|  | *Lishizhenia* | 0.00 | 0.00 | 0.00 | 0.02 |
|  | *Lutibacter* | 0.05 | 0.00 | 0.00 | 0.00 |
|  | *Meniscus* | 0.43 | 0.05 | 0.00 | 0.00 |
|  | *Muricauda* | 0.00 | 0.00 | 0.03 | 0.03 |
|  | *Odoribacter* | 0.00 | 0.00 | 0.26 | 0.00 |
|  | *Ohtaekwangia* | 0.00 | 0.23 | 0.00 | 0.09 |
|  | *Owenweeksia* | 0.00 | 0.09 | 0.02 | 0.03 |
|  | *Paludibacter* | 0.02 | 0.00 | 0.00 | 0.00 |
|  | *Parabacteroides* | 0.05 | 0.12 | 0.02 | 0.00 |
|  | *Pedobacter* | 0.00 | 0.09 | 0.00 | 0.00 |
|  | *Petrimonas* | 0.05 | 0.00 | 0.00 | 0.02 |
|  | *Prevotella* | 0.00 | 0.00 | 0.11 | 0.00 |
|  | *Prolixibacter* | 0.40 | 0.00 | 0.03 | 0.03 |
|  | *Proteiniphilum* | 0.02 | 0.00 | 0.00 | 0.00 |
|  | *Rikenella* | 0.06 | 0.00 | 0.00 | 0.00 |
|  | *Salisaeta* | 0.00 | 0.02 | 0.00 | 0.00 |
|  | *Tannerella* | 0.00 | 0.00 | 0.25 | 0.00 |
|  | *Terrimonas* | 0.00 | 0.42 | 0.00 | 0.00 |
|  | *Wandonia* | 0.00 | 0.02 | 0.00 | 0.00 |
| *BRC1* | *BRC1_genera_incertae_sedis* | 0.06 | 0.00 | 0.00 | 0.00 |
| *Chlamydiae* | *Parachlamydia* | 0.00 | 0.03 | 0.00 | 0.02 |
| *Chlorobi* | *Chlorobium* | 0.03 | 0.00 | 0.00 | 0.00 |
|  | *Ignavibacterium* | 0.00 | 0.17 | 0.37 | 0.54 |
| *Chloroflexi* | *Bellilinea* | 0.12 | 0.26 | 0.05 | 0.02 |
|  | *Caldilinea* | 0.57 | **5.95** | **3.08** | **3.28** |
| *Chloroflexi* | *Dehalogenimonas* | 0.08 | 0.03 | 0.02 | 0.00 |
|  | *Ktedonobacter* | 0.00 | 0.11 | 0.00 | 0.02 |
|  | *Levilinea* | 0.14 | 0.02 | 0.00 | 0.00 |
|  | *Longilinea* | 0.15 | 1.13 | 0.02 | 0.08 |
|  | *Sphaerobacter* | **1.00** | **2.47** | **2.46** | **1.85** |
| *Cyanobacteria/*  *Chloroplast* | *Bacillariophyta* | 0.00 | 0.26 | 0.00 | 0.00 |
| *Deinococcus-Thermus* | *Meiothermus* | 0.03 | 0.00 | 0.00 | 0.00 |
|  | *Truepera* | 0.05 | 0.39 | 0.46 | 0.63 |
| *Fibrobacteres* | *Fibrobacter* | 0.02 | 0.00 | 0.00 | 0.00 |
| *Firmicutes* | *Acetivibrio* | 0.02 | 0.00 | 0.00 | 0.00 |
|  | *Acetoanaerobium* | 0.02 | 0.02 | 0.00 | 0.00 |
|  | *Acetobacterium* | 0.00 | 0.09 | 0.02 | 0.00 |
|  | *Acidaminobacter* | 0.03 | 0.00 | 0.00 | 0.00 |
|  | *Acidaminococcus* | 0.00 | 0.02 | 0.00 | 0.00 |
|  | *Alkalibacillus* | 0.03 | 0.03 | 0.00 | 0.00 |
|  | *Alkalibacter* | 0.32 | 0.02 | 0.00 | 0.00 |
|  | *Alkalibacterium* | 0.02 | 0.00 | 0.00 | 0.00 |
|  | *Anaerofustis* | 0.03 | 0.05 | 0.00 | 0.00 |
|  | *Anaerostipes* | 0.00 | 0.00 | 0.05 | 0.00 |
|  | *Anaerotruncus* | 0.00 | 0.00 | 0.02 | 0.00 |
|  | *Anaerovorax* | 0.39 | 0.00 | 0.00 | 0.02 |
|  | *Aneurinibacillus* | 0.02 | 0.00 | 0.00 | 0.00 |
|  | *Anoxybacillus* | 0.00 | 0.02 | 0.00 | 0.00 |
|  | *Bacillus* | 0.03 | 0.00 | 0.00 | 0.00 |
|  | *Cellulosilyticum* | 0.00 | 0.00 | 0.02 | 0.00 |
|  | *Clostridium III* | 0.05 | 0.00 | 0.00 | 0.00 |
|  | *Clostridium IV* | 0.00 | 0.02 | 0.03 | 0.00 |
|  | *Clostridium sensu stricto* | 0.11 | 0.05 | 0.02 | 0.00 |
|  | *Clostridium XI* | 0.03 | 0.08 | 0.02 | 0.00 |
|  | *Clostridium XlVa* | 0.00 | 0.03 | 0.15 | 0.00 |
|  | *Clostridium XlVb* | 0.00 | 0.00 | 0.02 | 0.00 |
|  | *Cryptanaerobacter* | 0.08 | 0.00 | 0.00 | 0.00 |
|  | *Dethiobacter* | 0.02 | 0.00 | 0.00 | 0.00 |
|  | *Dethiosulfatibacter* | 0.06 | 0.00 | 0.00 | 0.00 |
|  | *Enterococcus* | 0.06 | 0.08 | 0.03 | 0.02 |
|  | *Eubacterium* | 0.02 | 0.02 | 0.00 | 0.00 |
|  | *Facklamia* | 0.02 | 0.00 | 0.03 | 0.03 |
|  | *Flavonifractor* | 0.03 | 0.00 | 0.02 | 0.00 |
|  | *Fusibacter* | 0.00 | 0.03 | 0.00 | 0.00 |
|  | *Gelria* | 0.02 | 0.00 | 0.00 | 0.00 |
|  | *Geosporobacter* | 0.02 | 0.00 | 0.00 | 0.00 |
|  | *Halanaerobium* | 0.03 | 0.02 | 0.00 | 0.00 |
| *Firmicutes* | *Lachnospiracea_incertae_sedis* | 0.00 | 0.00 | 0.08 | 0.00 |
|  | *Lactobacillus* | 0.00 | 0.00 | **3.34** | 0.00 |
|  | *Lactococcus* | 0.00 | 0.03 | 0.00 | 0.00 |
|  | *Marvinbryantia* | 0.00 | 0.00 | 0.03 | 0.00 |
|  | *Oscillibacter* | 0.02 | 0.00 | 0.12 | 0.00 |
|  | *Papillibacter* | 0.03 | 0.00 | 0.00 | 0.00 |
|  | *Paralactobacillus* | 0.00 | 0.00 | 0.03 | 0.00 |
|  | *Pasteuria* | 0.00 | 0.02 | 0.02 | 0.00 |
|  | *Pelospora* | 0.05 | 0.00 | 0.00 | 0.00 |
|  | *Pelotomaculum* | 0.06 | 0.00 | 0.00 | 0.00 |
|  | *Peptoniphilus* | 0.02 | 0.00 | 0.00 | 0.00 |
|  | *Phascolarctobacterium* | 0.03 | 0.14 | 0.00 | 0.00 |
|  | *Proteiniclasticum* | 0.57 | 0.00 | 0.09 | 0.12 |
|  | *Proteocatella* | 0.00 | 0.02 | 0.00 | 0.00 |
|  | *Pseudoflavonifractor* | 0.00 | 0.00 | 0.09 | 0.00 |
|  | *Pseudoramibacter* | 0.00 | 0.03 | 0.00 | 0.00 |
|  | *Roseburia* | 0.00 | 0.02 | 0.00 | 0.00 |
|  | *Saccharofermentans* | 0.40 | 0.02 | 0.00 | 0.00 |
|  | *Sedimentibacter* | 0.03 | 0.00 | 0.00 | 0.03 |
|  | *Soehngenia* | 0.03 | 0.00 | 0.00 | 0.00 |
|  | *Sporobacter* | 0.02 | 0.00 | 0.00 | 0.00 |
|  | *Streptococcus* | 0.00 | 0.05 | 0.03 | 0.00 |
|  | *Syntrophomonas* | 0.43 | 0.00 | 0.00 | 0.00 |
|  | *Syntrophothermus* | 0.00 | 0.02 | 0.00 | 0.00 |
|  | *Tissierella* | 0.54 | 0.03 | 0.02 | 0.05 |
|  | *Trichococcus* | 0.22 | 0.28 | 0.00 | 0.00 |
|  | *Turicibacter* | 0.06 | 0.05 | 0.00 | 0.02 |
|  | *Veillonella* | 0.00 | 0.08 | 0.00 | 0.00 |
| *Gemmatimonadetes* | *Gemmatimonas* | 0.03 | 0.32 | 0.20 | 0.19 |
| *Nitrospira* | *Nitrospira* | 0.00 | 0.80 | 0.00 | 0.00 |
| *OD1* | *OD1_genera_incertae_sedis* | 0.49 | 0.53 | **1.81** | **2.38** |
| *Planctomycetes* | *Gemmata* | 0.00 | 0.06 | 0.00 | 0.00 |
|  | *Isosphaera* | 0.02 | 0.15 | 0.02 | 0.03 |
|  | *Phycisphaera* | 0.00 | **1.58** | **1.19** | **1.11** |
|  | *Rhodopirellula* | 0.00 | 0.00 | 0.15 | 0.15 |
|  | *Singulisphaera* | 0.00 | 0.46 | 0.23 | 0.60 |
| *Proteobacteria*  *Proteobacteria*  *Proteobacteria* | *Afipia* | 0.00 | 0.17 | 0.00 | 0.00 |
|  | *Altererythrobacter* | 0.02 | 0.06 | 0.02 | 0.06 |
|  | *Amaricoccus* | 0.02 | 0.02 | 0.03 | 0.00 |
|  | *Aminobacter* | 0.00 | 0.02 | 0.03 | 0.00 |
|  | *Amorphus* | 0.00 | 0.02 | 0.00 | 0.00 |
|  | *Aquamicrobium* | 0.02 | 0.12 | 0.02 | 0.00 |
|  | *Bauldia* | 0.00 | 0.06 | 0.03 | 0.02 |
|  | *Blastochloris* | 0.00 | 0.02 | 0.02 | 0.02 |
|  | *Blastomonas* | 0.00 | 0.00 | 0.02 | 0.00 |
|  | *Bosea* | 0.00 | 0.08 | 0.00 | 0.00 |
|  | *Bradyrhizobium* | 0.00 | 0.06 | 0.00 | 0.00 |
|  | *Brevundimonas* | 0.00 | 0.03 | 0.00 | 0.00 |
|  | *Catellibacterium* | 0.00 | 0.06 | 0.00 | 0.00 |
|  | *Chelativorans* | 0.00 | 0.00 | 0.02 | 0.00 |
|  | *Citreicella* | 0.00 | 0.02 | 0.00 | 0.00 |
|  | *Defluviicoccus* | 0.02 | 0.26 | 0.31 | 0.43 |
|  | *Devosia* | 0.00 | 0.25 | 0.00 | 0.03 |
|  | *Dongia* | 0.02 | 0.11 | 0.00 | 0.00 |
|  | *Elioraea* | 0.00 | 0.03 | 0.08 | 0.06 |
|  | *Ensifer* | 0.00 | 0.14 | 0.09 | 0.03 |
|  | *Erythrobacter* | 0.00 | 0.00 | 0.00 | 0.02 |
|  | *Erythromicrobium* | 0.00 | 0.03 | 0.00 | 0.00 |
|  | *Filomicrobium* | 0.00 | 0.14 | 0.06 | 0.08 |
|  | *Fodinicurvata* | 0.00 | 0.02 | 0.00 | 0.00 |
|  | *Geminicoccus* | 0.02 | 0.08 | **1.02** | 0.68 |
|  | *Haematobacter* | 0.00 | 0.00 | 0.00 | 0.05 |
|  | *Hwanghaeicola* | 0.00 | 0.00 | 0.02 | 0.00 |
|  | *Hyphomicrobium* | 0.05 | **1.51** | **1.28** | **1.48** |
|  | *Hyphomonas* | 0.00 | 0.06 | 0.03 | 0.03 |
|  | *Mesorhizobium* | 0.00 | 0.39 | 0.05 | 0.06 |
|  | *Nitrobacter* | 0.00 | 0.29 | 0.06 | 0.06 |
|  | *Novosphingobium* | 0.05 | 0.19 | 0.03 | 0.03 |
|  | *Oceanibaculum* | 0.00 | 0.02 | 0.05 | 0.02 |
|  | *Oceanicola* | 0.00 | 0.00 | 0.06 | 0.03 |
|  | *Orientia* | 0.00 | 0.08 | 0.00 | 0.00 |
|  | *Paracoccus* | 0.03 | 0.17 | **1.08** | **1.27** |
|  | *Parvularcula* | 0.00 | 0.00 | 0.00 | 0.02 |
|  | *Pedomicrobium* | 0.00 | 0.02 | 0.00 | 0.00 |
|  | *Pelagibaca* | 0.00 | 0.02 | 0.00 | 0.02 |
|  | *Phaeobacter* | 0.00 | 0.00 | 0.00 | 0.02 |
|  | *Phenylobacterium* | 0.00 | 0.17 | 0.11 | 0.05 |
|  | *Pontibaca* | 0.09 | 0.00 | 0.00 | 0.03 |
|  | *Porphyrobacter* | 0.08 | 0.05 | 0.00 | 0.00 |
|  | *Pseudaminobacter* | 0.02 | 0.00 | 0.00 | 0.00 |
|  | *Pseudolabrys* | 0.00 | 0.06 | 0.02 | 0.02 |
|  | *Rhizobium* | 0.02 | 0.36 | 0.00 | 0.00 |
|  | *Rhizomicrobium* | 0.00 | 0.02 | 0.00 | 0.00 |
|  | *Rhodobacter* | 0.06 | 0.09 | 0.06 | 0.11 |
|  | *Rhodoplanes* | 0.00 | 0.12 | 0.00 | 0.00 |
|  | *Rhodovulum* | 0.00 | 0.05 | 0.03 | 0.02 |
|  | *Roseomonas* | 0.00 | 0.11 | 0.08 | 0.06 |
|  | *Rubribacterium* | 0.02 | 0.00 | 0.00 | 0.00 |
|  | *Sneathiella* | 0.00 | 0.03 | 0.02 | 0.00 |
|  | *Sphingobium* | 0.00 | 0.66 | 0.00 | 0.00 |
|  | *Sphingomonas* | 0.00 | 0.02 | 0.00 | 0.00 |
|  | *Sphingopyxis* | 0.00 | 0.22 | 0.00 | 0.02 |
|  | *Sphingosinicella* | 0.02 | 0.11 | 0.00 | 0.02 |
|  | *Stella* | 0.00 | 0.03 | 0.00 | 0.05 |
|  | *Tepidamorphus* | 0.00 | 0.03 | 0.00 | 0.00 |
|  | *Thioclava* | 0.00 | 0.00 | 0.02 | 0.00 |
|  | *Vasilyevaea* | 0.00 | 0.02 | 0.00 | 0.00 |
|  | *Xanthobacter* | 0.00 | 0.05 | 0.09 | 0.08 |
|  | *Acidovorax* | 0.03 | 0.29 | 0.00 | 0.00 |
|  | *Albidiferax* | 0.00 | 0.00 | 0.00 | 0.03 |
|  | *Aquabacterium* | 0.00 | 0.02 | 0.00 | 0.00 |
|  | *Azoarcus* | 0.00 | 0.06 | 0.36 | 0.46 |
|  | *Azohydromonas* | 0.00 | 0.11 | 0.00 | 0.02 |
|  | *Azovibrio* | 0.00 | 0.06 | 0.00 | 0.02 |
|  | *Brachymonas* | 0.03 | 0.00 | 0.00 | 0.00 |
|  | *Caldimonas* | 0.00 | 0.03 | 0.00 | 0.02 |
|  | *Comamonas* | 0.08 | 0.06 | 0.97 | 0.97 |
|  | *Curvibacter* | 0.00 | 0.02 | 0.00 | 0.06 |
|  | *Dechloromonas* | 0.02 | 0.09 | 0.00 | 0.00 |
|  | *Diaphorobacter* | 0.06 | 0.00 | 0.54 | 0.65 |
|  | *Georgfuchsia* | 0.00 | 0.03 | 0.00 | 0.00 |
|  | *Giesbergeria* | 0.00 | 0.02 | 0.00 | 0.00 |
|  | *Hydrogenophaga* | 0.05 | **1.07** | 0.66 | 0.91 |
|  | *Methyloversatilis* | 0.00 | 0.31 | 0.02 | 0.00 |
|  | *Nitrosomonas* | 0.00 | 0.34 | 0.11 | 0.22 |
|  | *Nitrosospira* | 0.00 | 0.20 | 0.15 | 0.15 |
|  | *Pandoraea* | 0.02 | 0.03 | 0.00 | 0.02 |
|  | *Polynucleobacter* | 0.02 | 0.00 | 0.00 | 0.00 |
|  | *Propionivibrio* | 0.00 | 0.03 | 0.00 | 0.00 |
|  | *Pusillimonas* | 0.02 | 0.00 | 0.00 | 0.02 |
|  | *Schlegelella* | 0.00 | 0.02 | 0.00 | 0.00 |
|  | *Shinella* | 0.00 | 0.48 | 0.00 | 0.02 |
|  | *Simplicispira* | 0.08 | 0.28 | 0.00 | 0.00 |
|  | *Sulfuritalea* | 0.00 | 0.03 | 0.00 | 0.00 |
|  | *Thauera* | 0.11 | **4.64** | **12.95** | **15.81** |
|  | *Thiobacillus* | 0.39 | 0.15 | 0.08 | 0.12 |
|  | *Thiobacter* | 0.00 | 0.06 | 0.02 | 0.03 |
|  | *Uliginosibacterium* | 0.00 | 0.02 | 0.02 | 0.00 |
|  | *Variovorax* | 0.00 | 0.08 | 0.00 | 0.00 |
|  | *Xenophilus* | 0.00 | 0.00 | 0.00 | 0.05 |
|  | *Bacteriovorax* | 0.00 | 0.03 | 0.00 | 0.00 |
|  | *Bdellovibrio* | 0.00 | 0.45 | 0.11 | 0.15 |
| *Proteobacteria* | *Byssovorax* | 0.00 | 0.32 | 0.00 | 0.00 |
|  | *Chondromyces* | 0.00 | 0.02 | 0.12 | 0.34 |
|  | *Desulfatibacillum* | 0.11 | 0.00 | 0.00 | 0.00 |
|  | *Desulfobacter* | **6.94** | 0.08 | 0.00 | 0.02 |
|  | *Desulfobulbus* | 0.96 | 0.00 | 0.00 | 0.00 |
|  | *Desulfococcus* | 0.08 | 0.03 | 0.02 | 0.00 |
|  | *Desulfomicrobium* | **1.70** | 0.03 | 0.00 | 0.02 |
|  | *Desulforhabdus* | 0.00 | 0.00 | 0.00 | 0.02 |
|  | *Desulfosarcina* | 0.15 | 0.03 | 0.00 | 0.00 |
|  | *Desulfotignum* | 0.00 | 0.00 | 0.02 | 0.00 |
|  | *Desulfovermiculus* | 0.00 | 0.02 | 0.00 | 0.00 |
|  | *Desulfovibrio* | 0.03 | 0.00 | 0.02 | 0.02 |
|  | *Desulfovirga* | 0.02 | 0.00 | 0.00 | 0.00 |
|  | *Desulfuromonas* | 0.43 | 0.00 | 0.00 | 0.02 |
|  | *Desulfuromusa* | 0.00 | 0.00 | 0.02 | 0.00 |
|  | *Kofleria* | 0.02 | 0.05 | 0.02 | 0.05 |
|  | *Nannocystis* | 0.00 | 0.03 | 0.03 | 0.00 |
|  | *Pelobacter* | 0.00 | 0.00 | 0.00 | 0.03 |
|  | *Peredibacter* | 0.00 | 0.03 | 0.00 | 0.00 |
|  | *Phaselicystis* | 0.00 | 0.05 | 0.02 | 0.11 |
|  | *Smithella* | 0.23 | 0.00 | 0.00 | 0.00 |
|  | *Syntrophorhabdus* | 0.19 | 0.00 | 0.00 | 0.00 |
|  | *Syntrophus* | 0.02 | 0.00 | 0.00 | 0.00 |
|  | *Vampirovibrio* | 0.02 | 0.19 | 0.91 | **1.55** |
|  | *Arcobacter* | 0.08 | 0.00 | 0.03 | 0.05 |
|  | *Sulfurospirillum* | 0.00 | 0.00 | 0.00 | 0.02 |
|  | *Sulfurovum* | 0.03 | 0.00 | 0.00 | 0.00 |
|  | *Aquicella* | 0.00 | 0.05 | 0.00 | 0.00 |
|  | *Aquimonas* | 0.02 | 0.03 | 0.00 | 0.02 |
|  | *Arenimonas* | 0.11 | 0.02 | 0.00 | 0.00 |
|  | *Congregibacter* | 0.00 | 0.00 | 0.00 | 0.02 |
|  | *Dokdonella* | 0.00 | 0.00 | 0.00 | 0.02 |
|  | *Haliea* | 0.00 | 0.06 | 0.12 | 0.06 |
|  | *Halochromatium* | 0.00 | 0.00 | 0.20 | 0.25 |
|  | *Legionella* | 0.00 | 0.08 | 0.00 | 0.00 |
|  | *Lysobacter* | 0.02 | 0.02 | 0.00 | 0.00 |
|  | *Marichromatium* | 0.00 | 0.79 | 0.03 | 0.14 |
|  | *Methylomonas* | 0.00 | 0.02 | 0.00 | 0.00 |
|  | *Porticoccus* | 0.00 | 0.02 | 0.00 | 0.00 |
|  | *Pseudofulvimonas* | 0.02 | 0.09 | 0.00 | 0.00 |
|  | *Rudaea* | 0.00 | 0.00 | 0.03 | 0.02 |
|  | *Steroidobacter* | 0.05 | 0.62 | 0.08 | 0.19 |
|  | *Thioalkalibacter* | 0.00 | 0.00 | 0.02 | 0.00 |
|  | *Thiocapsa* | 0.02 | 0.05 | 0.08 | 0.03 |
| *Proteobacteria* | *Thiofaba* | 0.03 | 0.00 | 0.00 | 0.00 |
|  | *Thiohalocapsa* | 0.00 | 0.00 | 0.02 | 0.03 |
|  | *Thiohalophilus* | 0.00 | 0.00 | 0.02 | 0.00 |
|  | *Thioprofundum* | 0.00 | 0.02 | 0.08 | 0.06 |
|  | *Thiothrix* | 0.00 | 0.03 | 0.02 | 0.00 |
| *Spirochaetes* | *Spirochaeta* | 0.03 | 0.00 | 0.00 | 0.03 |
|  | *Treponema* | 0.03 | 0.00 | 0.00 | 0.00 |
| *SR1* | *SR1_genera_incertae_sedis* | 0.14 | 0.05 | 0.31 | 0.36 |
| *Synergistetes* | *Aminobacterium* | **11.47** | 0.12 | 0.00 | 0.05 |
|  | *Aminomonas* | **2.21** | 0.00 | 0.02 | 0.00 |
|  | *Cloacibacillus* | **2.98** | 0.12 | 0.00 | 0.02 |
|  | *Dethiosulfovibrio* | 0.06 | 0.00 | 0.02 | 0.00 |
|  | *Thermovirga* | **3.00** | 0.02 | 0.00 | 0.02 |
| *Tenericutes* | *Acholeplasma* | 0.06 | 0.03 | 0.00 | 0.00 |
|  | *Anaeroplasma* | 0.00 | 0.00 | 0.06 | 0.00 |
| *Thermotogae* | *Kosmotoga* | **5.75** | 0.03 | 0.00 | 0.02 |
|  | *Thermococcoides* | 0.23 | 0.00 | 0.00 | 0.00 |
| *TM7* | *TM7_genera_incertae_sedis* | 0.17 | **4.13** | 0.59 | 0.65 |
| *Verrucomicrobia* | *Opitutus* | 0.00 | 0.02 | 0.00 | 0.00 |
|  | *Prosthecobacter* | 0.00 | 0.29 | 0.03 | 0.03 |
|  | *Spartobacteria_genera_incertae_sedis* | 0.00 | 0.03 | 0.00 | 0.00 |
|  | *Subdivision3_genera_incertae_sedis* | 0.00 | 0.36 | 0.00 | 0.00 |
|  | *Subdivision5_genera_incertae_sedis* | 0.00 | 0.02 | 0.00 | 0.00 |
|  | *Verrucomicrobium* | 0.00 | 0.02 | 0.00 | 0.00 |
| *WS3* | *WS3_genera_incertae_sedis* | 0.19 | **1.14** | **5.42** | **5.08** |
