# Supplementary material for: Abundance and Diversity of Bacterial Nitrifiers and Denitrifiers and Their Functional Genes in Tannery Wastewater Treatment Plants Revealed by High-Throughput Sequencing
Source: PLoS One. 2014 Nov 24;9(11):e113603. doi: 10.1371/journal.pone.0113603 (PMC4242629; doi:10.1371/journal.pone.0113603)
Supplement: Table S5 — COGs related to denitrification and nitrogen fixation identified in the four activated sludge metagenomes. (DOCX) [file pone.0113603.s014.docx]

**Table S5** COGs **related to denitrification and nitrogen fixation identified in the four activated sludge metagenomes.**

| COG | Annotation | Number of hits | | | |
| --- | --- | --- | --- | --- | --- |
|  |  | A-A | A-O | B-D | B-O |
| COG1140 | Nitrate reductase beta subunit | 664 | 1501 | 879 | 978 |
| COG1348 | Nitrogenase subunit NifH (ATPase) | 253 | 192 | 99 | 121 |
| COG2223 | Nitrate/nitrite transporter | ND | 1581 | ND | ND |
| COG2710 | Nitrogenase molybdenum-iron protein, alpha and beta chains | ND | 829 | ND | 521 |
| COG3005 | Nitrate/TMAO reductases, membrane-bound tetraheme cytochrome c subunit | ND | 278 | ND | ND |
| COG3256 | Nitric oxide reductase large subunit | ND | 1754 | 1492 | 1637 |
| COG3420 | Nitrous oxidase accessory protein | ND | ND | NA | 399 |
| COG4263 | Nitrous oxide reductase | 104 | 741 | 612 | 845 |
| COG5013 | Nitrate reductase alpha subunit | 1297 | 3095 | 1822 | 1841 |
