# Supplementary material for: Abundance and Diversity of Bacterial Nitrifiers and Denitrifiers and Their Functional Genes in Tannery Wastewater Treatment Plants Revealed by High-Throughput Sequencing
Source: PLoS One. 2014 Nov 24;9(11):e113603. doi: 10.1371/journal.pone.0113603 (PMC4242629; doi:10.1371/journal.pone.0113603)
Supplement: Table S6 — Composition of nitrifying bacteria (AOB and NOB) in the four sludge samples revealed by 454 pyrosequencing. (DOCX) [file pone.0113603.s015.docx]

**Table S6 Composition of nitrifying bacteria (AOB and NOB) in the four sludge samples revealed by 454 pyrosequencing.**

| Sample | Number of  clean reads | AOB | | | | NOB | | | |
| --- | --- | --- | --- | --- | --- | --- | --- | --- | --- |
|  |  | *Nitrosomonas* | | *Nitrosospira* | | *Nitrobacter* | | *Nitrospira* | |
|  |  | Reads | % | Reads | % | Reads | % | Reads | % |
| A-A | 6471 | — | — | — | — | — | — | — | — |
| A-O | 6471 | 22 | 0.34 | 13 | 0.20 | 19 | 0.29 | 52 | 0.80 |
| B-D | 6471 | 7 | 0.11 | 10 | 0.15 | 4 | 0.06 | — | — |
| B-O | 6471 | 14 | 0.22 | 10 | 0.15 | 4 | 0.06 | — | — |
