# Supplementary material for: Abundance and Diversity of Bacterial Nitrifiers and Denitrifiers and Their Functional Genes in Tannery Wastewater Treatment Plants Revealed by High-Throughput Sequencing
Source: PLoS One. 2014 Nov 24;9(11):e113603. doi: 10.1371/journal.pone.0113603 (PMC4242629; doi:10.1371/journal.pone.0113603)
Supplement: Table S7 — Potential denitrifying genera detected in the four sludge samples through 454 pyrosequencing. The reads number was calculated by normalizing the total pyrosequencing reads to 6,471 for each sample. (DOCX) [file pone.0113603.s016.docx]

**Table S7** **Potential denitrifying genera detectedd in the four tannery activated sludge samples through 454 pyrosequencing.** The reads number was calculated by normalizing the total pyrosequencing reads to 6,471 for each sample.

| Potential denitrifying genera | Number of Reads | | | |
| --- | --- | --- | --- | --- |
|  | A-A | A-O | B-D | B-O |
| *Acidovorax* | 2 | 19 | 0 | 0 |
| *Azoarcus* | 0 | 4 | 23 | 30 |
| *Bacillus* | 2 | 0 | 0 | 0 |
| *Brachymonas* | 2 | 0 | 0 | 0 |
| *Bradyrhizobium* | 0 | 4 | 0 | 0 |
| *Comamonas* | 5 | 4 | 63 | 63 |
| *Corynebacterium* | 0 | 3 | 0 | 1 |
| *Flavobacterium* | 1 | 1 | 4 | 0 |
| *Hyphomicrobium* | 3 | 98 | 83 | 96 |
| *Nitrobacter* | 0 | 19 | 4 | 4 |
| *Nitrosomonas* | 0 | 22 | 7 | 14 |
| *Paracoccus* | 2 | 11 | 70 | 82 |
| *Rhizobium* | 1 | 23 | 0 | 0 |
| *Rhodobacter* | 4 | 6 | 4 | 7 |
| *Rhodoplanes* | 0 | 8 | 0 | 0 |
| *Thauera* | 7 | 300 | 838 | 1023 |
| *Thiobacillus* | 25 | 10 | 5 | 8 |
